# "Missing" G x E Variation Controls Flowering Time in *Arabidopsis thaliana*

**DOI:** 10.1371/journal.pgen.1005597

**Published:** 2015-10-16

**Authors:** Eriko Sasaki, Pei Zhang, Susanna Atwell, Dazhe Meng, Magnus Nordborg

**Affiliations:** 1 Gregor Mendel Institute, Austrian Academy of Sciences, Vienna Biocenter (VBC), Vienna, Austria; 2 Molecular and Computational Biology, University of Southern California, Los Angeles, California, United States of America; Georgia Institute of Technology, UNITED STATES

## Abstract

Understanding how genetic variation interacts with the environment is essential for understanding adaptation. In particular, the life cycle of plants is tightly coordinated with local environmental signals through complex interactions with the genetic variation (G x E). The mechanistic basis for G x E is almost completely unknown. We collected flowering time data for 173 natural inbred lines of *Arabidopsis thaliana* from Sweden under two growth temperatures (10°C and 16°C), and observed massive G x E variation. To identify the genetic polymorphisms underlying this variation, we conducted genome-wide scans using both SNPs and local variance components. The SNP-based scan identified several variants that had common effects in both environments, but found no trace of G x E effects, whereas the scan using local variance components found both. Furthermore, the G x E effects appears to be concentrated in a small fraction of the genome (0.5%). Our conclusion is that G x E effects in this study are mostly due to large numbers of allele or haplotypes at a small number of loci, many of which correspond to previously identified flowering time genes.

## Author Summary

Many traits are influenced by genetic variation in interaction with the environment, so called G x E variation. In agriculture, for example, different varieties are optimal in different environments. In evolution, G x E is also crucial for local adaptation. Identifying the genes underlying G x E has proven extremely challenging, however. Using a collection of inbred lines of the model plant *Arabidopsis thaliana*, we meausured flowering time under two temperature regimes, and scanned the genome for polymorphisms responsible for variation in this trait. Although most of the variation is due to G x E, genome-wide scans using SNPs only revealed direct genetic effects (G), and failed to reveal any significant G x E associations. In contrast, scanning the genome using local windows of polymorphism suggested that almost all the observed variation can be explained by 2% of the genome. Previously identified flowering time genes are strongly overrepresented in these regions, and our results are compatible with a model under which G x E is mainly due to many alleles at a relatively small number of loci.

## Introduction

The transition from vegetative to reproductive growth is a key developmental step in the life cycle of higher plants, and its timing is tightly regulated by both genes and environment, often in an interactive manner, so that the effect of genetic variants depends on the environment [1, 2]. Such genotype by environment interactions (G x E) have long been of interest to quantitative geneticists, as they are crucial for local adaptation [3, 4] and for improving agricultural yield. In particular, understanding G x E variation is considered essential for predicting the effects of climate change on ecology and agriculture [2, 5].

Analytically, G x E can be described in terms of “reaction norms” as genetic variation in the phenotypic response to the environment [2]. The phenotypic variation can be decomposed into genetic effects that are the same across environments (G), effects that are different across environments (G x E), and non-genetic environmental effects (E). Many approaches have been proposed to identify loci contributing to G x E variation [2, 6]. In the context of genome-wide association studies (GWAS), Korte *et al*. [7] proposed a multi-trait mixed model (MTMM) that can also be used to study G x E [2, 5, 7].

Attempts to map G x E variation, whether using classical linkage mapping or GWAS [4, 5, 8–10], have generally revealed loci explaining only a small fraction of the G x E variation. The most likely explanation for this “missing” G x E heritability is that the underlying genetic architecture involves either rare alleles of relatively large effect [2], or large numbers of polymorphisms of small effect [5, 8, 9].

Here we present a GWAS for flowering time at two temperatures (10°C and 16°C; see Methods) in a population of 173 *A. thaliana* lines from Sweden [11] (S1 Fig, S1 Table). Our goal was twofold: first, we wanted to investigate our ability to map polymorphisms responsible for G x E interactions; second, we wanted to characterize the main determinants of flowering time variation in Sweden, because although many GWAS have mapped genes responsible for flowering time variation in *A. thaliana* [5, 12–15], this has almost always been done in global samples, and there is reason to believe that the relatively small number of significant associations in these attempts is due to excessive genetic heterogeneity in these samples. The genetics of flowering time in local samples could be simpler, increasing the power of GWAS [12].

## Results

### Reaction norms and G x E

**Fig 1:**
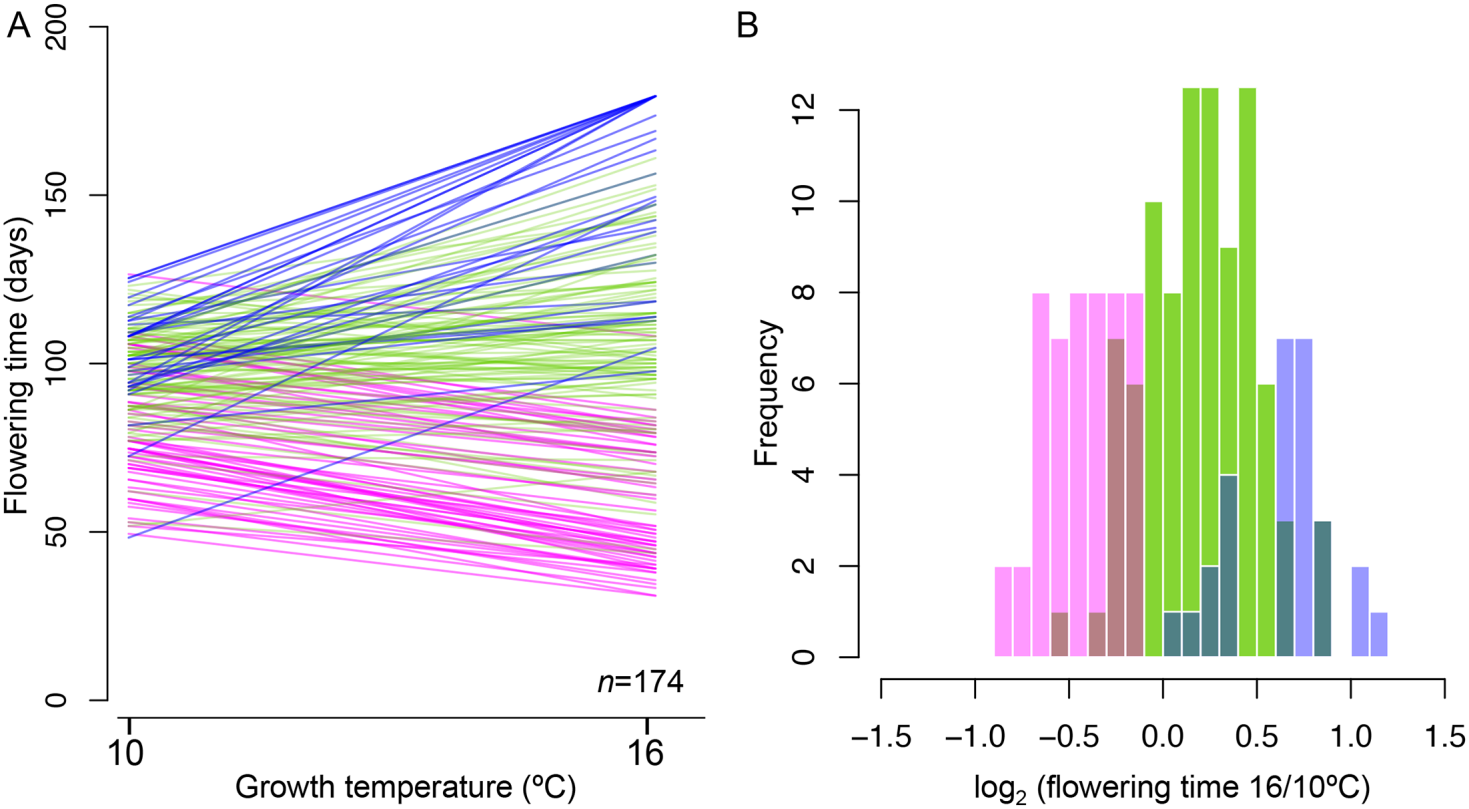
Reaction norms for flowering time at 10°C and 16°C in 173 Swedish lines (plus Col-0). A. Flowering time was significantly accelerated in 51 lines (shown in magenta), significantly decelerated in 28 lines (blue), and not significantly affected in 95 lines (green). B. Histogram of the ratio of flowering times using the same color scheme as in A.

The increase in growing temperature from 10°C to 16°C had a dramatic effect on flowering behavior, significantly accelerating flowering in 29% of the lines, significantly decelerating flowering in 16% of the lines, and generally increasing the variance both within and between lines (*t*-test, *q*-value < 0.01; Fig 1; S1–S2 Tables). Broad-sense heritabilities (*H* ^2^) were extremely high (over 90%) at both temperatures (albeit significantly lower at 16°C, *p* < 0.01), demonstrating strong genetic effects, in agreement with published results (Table 1) [12, 16, 17]. We partitioned the variance in flowering time using a model with four components: genotype (G, the variance attributable to genome-wide relatedness), environment (E), G x E, and noise (see Methods). This analysis revealed massive G x E effects. The G x E effects are largely due to the differences in the reaction norm between the subsets in Fig 1. For example, 67.9% of the variation among lines with accelerated flowering is due to direct genetic effects (Table 2).

**Table 1:** Broad-sense heritability of flowering time in both temperatures, for the full sample and separately for the subsets of lines that responded differently to the change in temperature. “Accelerated flowering”, “decelerated flowering”, and “no response” correspond to magenta, blue, and green lines in Fig 1, respectively. *N* is total number of individuals and *μ* is average of flowering time in the group.

	*H* ^2^	df	*N*	*μ*
Whole population				
10°C	0.97	173	549	92.8
16°C	0.91	173	612	99.0
Accelerated flowering				
10°C	0.98	50	162	81.3
16°C	0.95	50	211	60.8
Decelerated flowering				
10°C	0.97	27	97	102.2
16°C	0.87	27	74	152.7
No response				
10°C	0.95	94	290	96.12
16°C	0.74	94	327	109.78

**Table 2:** Genetic and environmental effects on flowering time variation. “Accelerated flowering”, “decelerated flowering”, and “no response” correspond to magenta, blue, and green lines in Fig 1, respectively.

	Single-trait (%)		Multi-trait (%)			
	G_10°*C*_	G_16°*C*_	G	G x E	E	noise
Whole population	99.95	99.99	28.43	65.94	5.62	10^−4^
Accelerated flowering	99.98	99.99	67.90	5.79	26.30	10^−4^
Decelerated flowering	99.91	99.98	8.35	24.31	67.33	10^−4^
No response	86.75	70.16	36.29	29.1	14.8	19.8

### GWAS of G x E

We attempted to map the polymorphisms responsible for the G x E effect using genome-wide association using a mixed model that allows multiple correlated traits (MTMM [7]). Three different association tests were carried out: a “full SNP test” that compares a full model including the effect of marker genotype and its interaction with environment against a model with no (fixed) SNP effect; “common SNP effect test” that compare a model with genetic marker (a genetic model) against no SNP effect, and; “interaction (G_SNP_ x E) effect test” that compares the full model against the genetic model [7]. In agreement with previous results, MTMM appeared to correct for confounding population structure well, whereas a standard multi-linear regression model (MLR) produced massively skewed *p*-values (S2 Fig).

**Fig 2:**
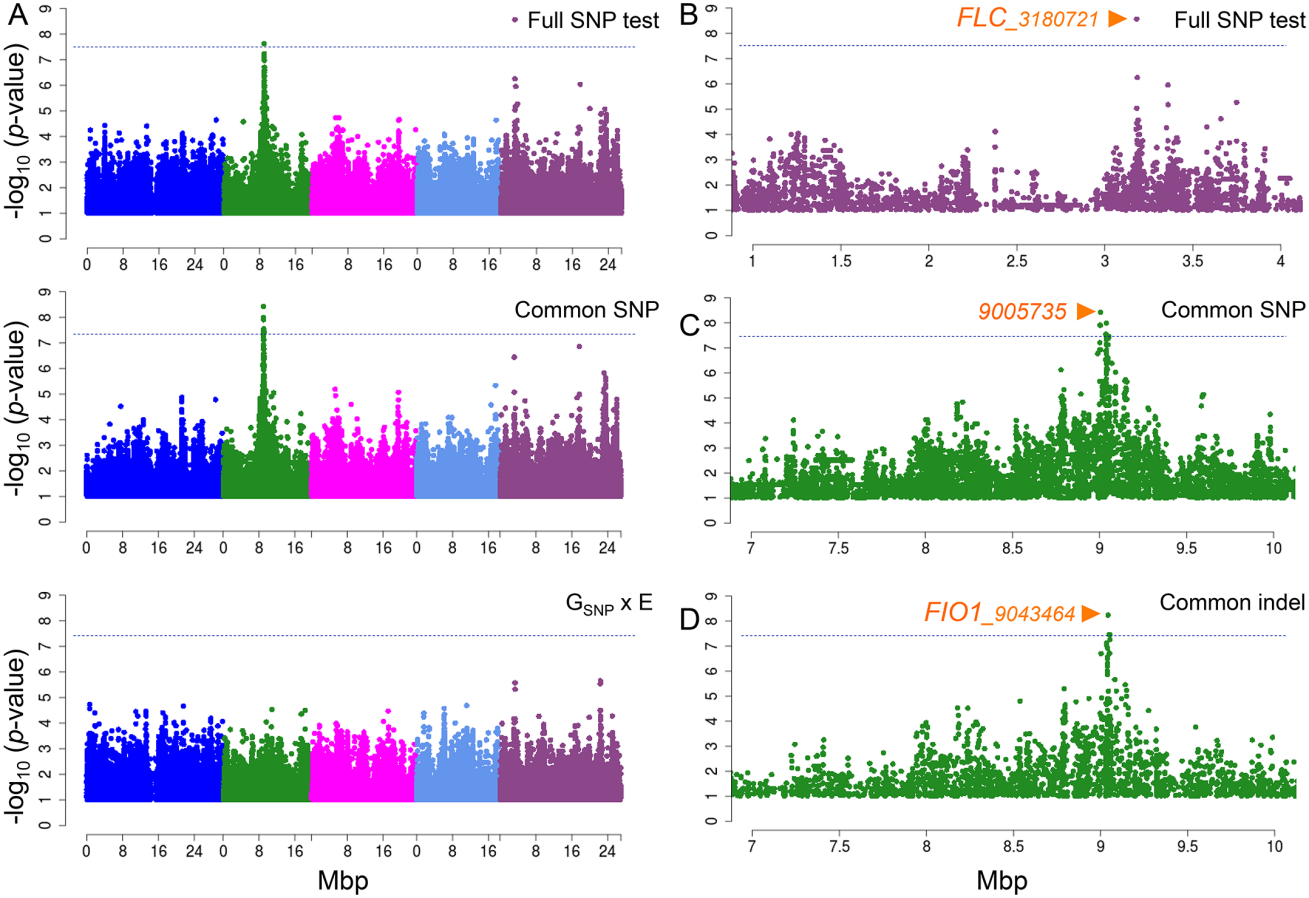
Manhattan plots of GWAS results for flowering time at 10°C and 16°C using MTMM. A. From top to bottom, results for full SNP, common SNP effect, and G_SNP_ x E effect tests. B. Zoom-in on chromosome 5 peak from full SNP test. C. Zoom-in on chromosome 2 peak from common effect by SNP markers, and D. by indel markers. Orange arrows show position of the strongest association in the peak. Horizontal dashed lines show 5% genome-wide significance thresholds after Bonferroni-correction.

The full SNP test identified two peaks with genome-wide significance (Fig 2A). The strongest association was centered around position 3,180,721 on chromosome 5, in the promoter region of the well-known flowering regulator *FLOWERING LOCUS C* (*FLC*) (Fig 2B), which has previously been shown to play a major role in natural variation for flowering time, but has generally been difficult to map using GWAS [5, 12, 13], presumably because of extensive genetic heterogeneity [18, 19]. Interestingly, the *FLC* peak can be seen using both the common SNP and the G_SNP_ x E effect tests, but was significant in neither, suggest that it has a weak G_SNP_ x E effect as well as a weak common SNP effect.

The behavior of the second strong association is very different. This association, centered on position 9,005,735 on chromosome 2, is more significant under the common SNP effect test, and is not present under the G_SNP_ x E effect test, suggesting that the polymorphism has the same effect in both temperatures. The peak is quite broad (Fig 2C) and contains approximately 13 genes, none of which are known to be involved in regulating flowering time. However, one of them, *FIONA1* (*FIO1*), is related to the circadian clock, and the null mutant shows early flowering [20]. Furthermore, GWAS using indel markers identified the most significant association (*p*-value = 2.97E-08; Fig 2D) as a insertion of two nucleotides in the 9th (last) exon of *FIO1*, which would result in a frameshift, however, this exon appears not to be present in mRNA-seq data from leaves [21], and appears to be specific to *A. thaliana*. A stop codon is found 26-amino acids upstream of the insertion in the closely related *Arabidopsis lyrata* and *Capsella rubella*. The putative frameshift polymorphism is due to eight vs nine GA repeats, and is in strong linkage disequilibrium with several non-synonymous polymorphisms, which are slightly less strongly associated with flowering time (S3 Fig). Although definitive proof in the form of transgenic experiments (allele swapping) is missing, polymorphism in *FIO1* is a strong candidate for the major common effect on chromosome 2. The common SNP effect test revealed no further significant associations, and the G_SNP_ x E effect test revealed no significant associations at all, despite the fact that G x E effects account for 66% of the phenotypic variance (Fig 2, Table 2).

### Enrichment of *a priori* candidates

Our GWAS identified two associations with genome-wide significance, one of which corresponds to a clear *a priori* candidate (*FLC*). Given that the number of *a priori* candidates (genes known to be involved in flowering time) is on the order of a percent of total genes (S3 Table), one out of two is obviously more than expected by chance. To investigate whether there is an overrepresentation of *a priori* candidates among associations that do not reach genome-wide significance as well, we calculated the enrichment as a function of significance threshold [12]. Because an association that is significant at a certain level will generally be surrounded by many SNPs that are less strongly associated (giving rise to a peak of association), we calculated enrichment at a given level after removing all peaks (defined as 30 kbp windows) containing SNPs that were already significant using a more stringent threshold.

**Fig 3:**
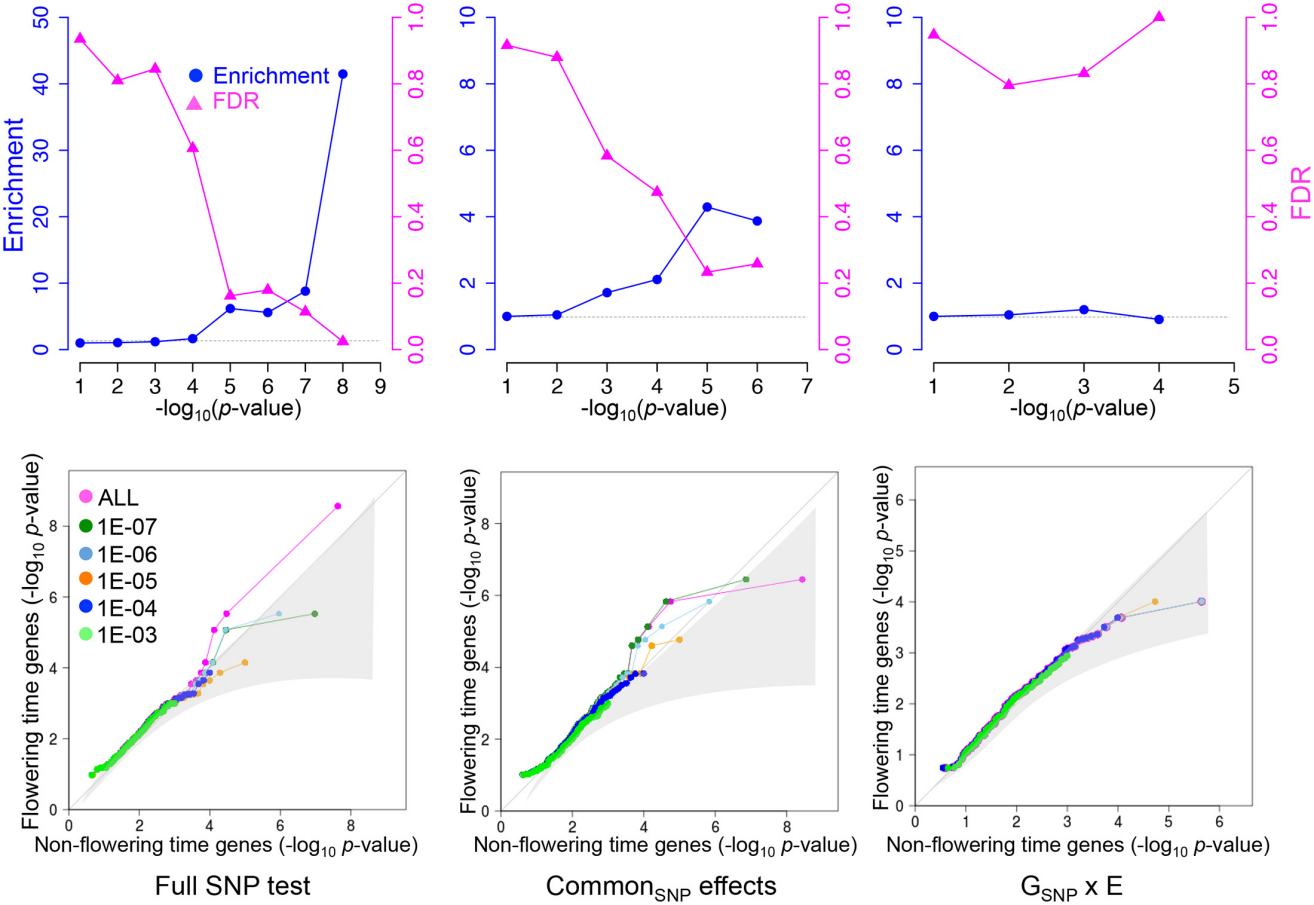
Enrichment for *a priori* flowering time candidates in MTMM. Top row: enrichment and FDR (upper bound among *a priori* candidates. The horizontal dashed lines at 1 corresponds to no enrichment. Bottom row: Quantile-quantile plots comparing the distribution of *p*-values in windows containing *a priori* candidates with windows that do not. The different curves show results after removing windows significant using more stringent thresholds (see text). The shaded region corresponds to a 95% confidence interval. See Methods for details.

For the full SNP test, a significant enrichment of *a priori* candidates persists as we increase the significance threshold (i.e., lower the stringency) to 10^−5^ (Fig 3). Although associations at this level are far from significant in the genome-wide sense, the enrichment of *a priori* candidates implies that the false-discovery rate (FDR) among these candidates is less than 20% [12]. Three *a priori* candidates were identified using this approach (Table 3): *FLC* (which also reaches genome-wide significance); *SHORT VEGETATIVE PHASE* (*SVP*), which mediates ambient temperature signaling by regulating *FLOWERING LOCUS T* (*FT*) [22], and has been shown to be involved in natural variation in other samples [23]; and *VERNALIZATION INSENSITIVE 3* (*VIN3*), which is involved in the epigenetic silencing of *FLC* during vernalization, but has hitherto not been identified in natural populations [20, 24]. Some of the associated SNPs were found in promoter regions (common SNP effects of *FLC*, *VIN3*). These SNPs are excellent candidates for being causal, and it seems likely that we simply lack the power to pick them up in a genome-wide scan. What the FDR is among the approximately 10 peaks that do not correspond to *a priori* candidates but are significant using the same threshold is not known (S4 Table).

**Table 3:** *A priori* candidates identified at FDR less than 20% by SNP association test.

		Full SNP test		Common SNP		G_SNP_ x E	
Gene name	Chr	Position	*p*-value (MAF)	Position	*p*-value (MAF)	Position	*p*-value (MAF)
*SVP*	2	9593397	2.96E-06 (25.9)	9593397	7.36E-06 (25.9)	9580035	4.8E-04 (32.8)
*FLC*	5	3180721	2.72E-09 (39.1)	3180721	3.58E-07 (39.1)	3184162	9.85E-05 (29.3)
*VIN3*	5	23249568	8.48E-06 (10.9)	23249568	1.47E-06 (10.9)	23249256	0.014 (19.5)

The results for the common SNP effect test were very similar to the full SNP test, and the same *a priori* candidates were identified (Fig 3, Table 3). However, the G_SNP_ x E effect test showed no evidence for significant enrichment at any *p*-value threshold, suggesting that if low power is the reason for the missing G x E associations, then the power is low indeed.

Finally, we note that if causal variants are strongly correlated with global relatedness, power to detect them may be greatly decreased [25, 26]. We therefore scanned for associations without correction for relatedness (using MLR), as well. The associations from such an analysis are of course extremely inflated, but it is possible to use the enrichment analysis described above, as it does not rely on well calibrated *p*-values (S2, S4 Figs). However, this approach identified only a subset of the candidate genes already identified using MTMM.

### Using local relatedness to improve power

Statistical power in GWAS may be decreased by allelic heterogeneity, which reduces the marginal contribution of individual polymorphisms at a genetic locus. One possible way around this is to consider the joint effect of all polymorphisms at a genetic locus using a mixed model. Instead of mapping individuals SNPs as fixed effects, we estimate the variance component that is due to local relatedness around each gene (using a 15 kbp window on each side of the coding region) and compare that to the variance component that is due to the rest of the genome [21]. We refer to these effects as “local” and “global”, respectively, and we also include environmental and G x E components.

Three different tests were carried out: a “full local test” that compares a full model, including local and global effects and their interactions with E, with a null model that does not include any local effect; a “common local effect test” that compares a local model that does not include a G_local_ x E with the null model, and; an “interaction (G_local_ x E) effect test” that compares the full model with the local model. For each test, log-likelihood ratios were calculated (see Methods).

**Fig 4:**
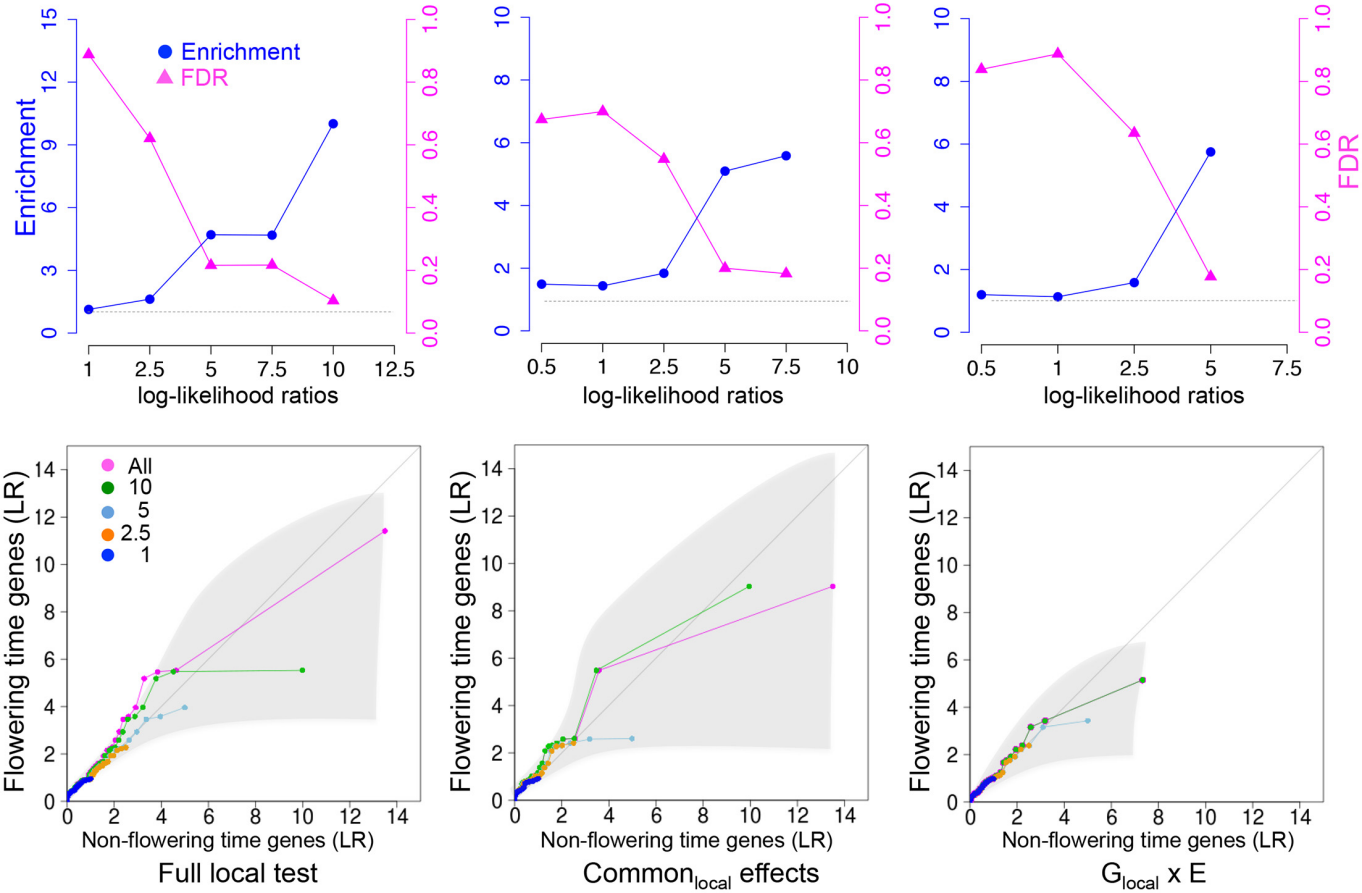
Enrichment for *a priori* flowering time candidates in using local variance component analysis. Top row: enrichment and FDR (upper bound among *a priori* candidates). The horizontal dashed lines at 1 corresponds to no enrichment. Bottom row: Quantile-quantile plots comparing the distribution of likelihood ratios in windows containing *a priori* candidates with windows that do not. The different curves shows result after removing windows significant using more stringent thresholds (see text). The shaded region corresponds to a 95% confidence interval. See Methods for details.

Result for the full local and the common local effect tests were strongly correlated with their corresponding GWAS results (presented above), especially for genes with reasonably strong association with flowering, while G_SNP_ x E and G_local_ x E showed much lower correlation (S5 Fig). Because the variance component likelihood ratios are not calibrated, it is difficult to say whether any particular effect is significant. However, we can assess this using overrepresentation of *a priori* candidates as for MTMM above. In all tests (full local, common local and G_local_ x E), a significant enrichment of *a priori* candidates exist for likelihood ratios of 5 or higher, for which FDR is less than 20% (Fig 4). Notably, this effect was observed for the G_local_ x E effect test as well, whereas G_SNP_ x E showed no evidence of overrepresentation (Fig 3). Thus the variance component analysis appears to capture G x E effects not captured by the marginal SNP GWAS.

A total of four flowering time genes showed significant peaks at the log-likelihood threshold of 5 (Table 4). *FLC* and *VIN3* showed high common local effect as well as common SNP effect, while *FPA*, an *FLC* suppressor in the autonomous pathway [28], showed up as a G_local_ x E locus. Furthermore, *CENTER CITY* (*CCT*) was significant in using the full local test. *CCT*, also known as *CRYPTIC PRECOCIOUS* (*CRP*), is a flowering regulator that acts as a promoter of *FT* and a suppressor of *FLC* [29, 30]. It is closely linked to the well-known flowering time locus *FRIGIDA* (*FRI*) and has previously been detected in GWAS [12].

**Table 4:** *A priori* candidates identified at 20% FDR by local association test.

ID	Start	End	Full local test	G_local_ (LR)	G_local_ x E (LR)	Candidate genes
14	AT2G43350	AT2G43410	**5.45**	0.04	**5.4**	*FPA*
25	AT4G00450	AT4G00590	**6.37**	4.57	3.31	*CCT*
30	AT5G10090	AT5G10260	**11.69**	**9.65**	4.1	*FLC*
38	AT5G57345	AT5G57410	**6.31**	**6.31**	0.1	*VIN3*

### The genomic architecture of associations

**Fig 5:**
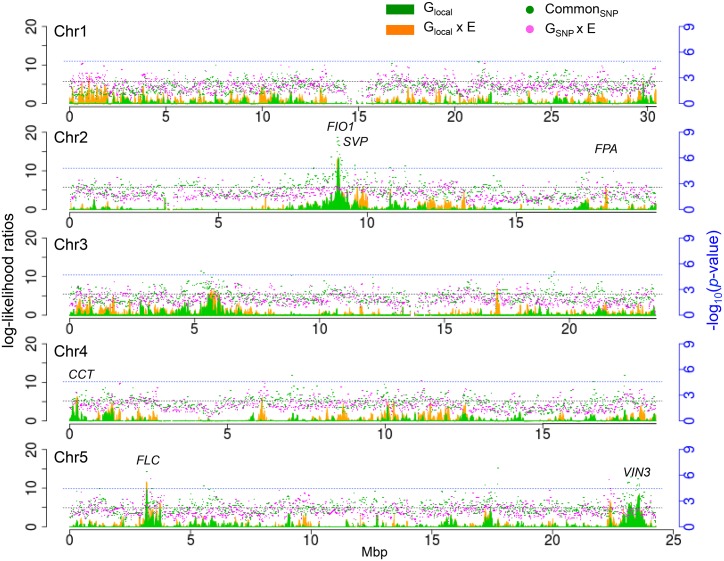
Genome-wide G and G x E effects for SNPs and well as local variance components. Dotted lines correspond to significance cut-offs of *p*-value = 10^−5^ for SNP associations (in blue) and log-likelihood ratios = 5 for variance components (in black).

Fig 5 shows the distribution of common (i.e., G) and G x E signals across the genome, for SNPs as well as for local variance components. The three highest peaks of G_local_ (S5 Table) overlap peaks of common G_SNP_ effect centered around *FIO1* on chromosome 2, and *FLC* on chromosome 5, and position 23,544,472 on chromosome 5. This overlap suggests that a small number of SNPs identified by MTMM might be responsible for the local variance components. Although there are no obvious flowering time candidates in the final region on chromosome 5, a recent study reported that gene in the region, *MULTICOPY SUPRESSOR OF IRA 1* (*MSI*; AT5G58230) delays the transition to flowering [31]. The most significant peak of G_local_ x E only was found at the top of chromosome 1 (963,400-1,053,719) and includes eight genes, none of which are known to be involved in flowering.

Finally, we consider the question of genetic architecture. For a Mendelian trait, all the phenotypic variation is due to a single locus, whereas for a truly Fisherian trait, the contribution of a genomic region should be proportional to its size (relative to the entire genome). Flowering time is clearly neither. As shown in Table 5, the 144 SNPs identified using MTMM (with the full SNP test using the 20% FDR defined in Fig 3) jointly explain 22% of the phenotypic variation as common (to both environments) genetic variation (G), and 31% as G x E variation. The remaining 3.7 million SNPs (of which 1 million have a minor allele frequency less than 0.1) explain only 6% as G and 35% as G x E. If we instead turn to the local variance components, the identified regions, comprising roughly 2% of the genome, explain 26% as G and 67% as G x E (randomly chosen regions explain on average at total of 7.5%; *p* = 0.001; S6 Fig), supporting the observation that the local variance component approach seems to have significantly greater power to capture G x E effects, but does not do better when it comes to common effects. Importantly, the local variance components explain essentially all the available genetic variation, and combining SNPs and local variance components yield almost no improvement (Table 5). It is also worth noting that the less than 10% of the identified regions that contain one of the *a priori* candidates explain almost 40% of the variation, a clearly significant overrepresentation (*p* = 0.001; S6 Fig).

**Table 5:** Summary of variance explained by SNPs identified using MTMM and VCA. Numbers in parenthesis are likelihood ratios (LR).

	G_sig._	G_sig._ x E	G_not_sig._	G_not_sig._ x E	E	Noise
MTMM						
All (144 SNPs)	21.53 (34.91)	31.27 (8.22)	6.45 (5.69)	34.90 (13.31)	3.76	2.14
*A priori* (9 SNPs)	9.63 (27.02)	7.50 (7.2)	18.83 (13.47)	58.52 (13.32)	5.51	0.01
VCA						
All local (2.0% of genome; 43,554 SNPs)	25.57 (27.38)	67.09 (21.36)	1.14 (0.06)	0.14 (0)	6.05	0.02
*A priori* (0.13% of genome; 3,101 SNPs)	11.03 (8.85)	30.76 (10.80)	17.18 (12.64)	35.47 (8.84)	5.55	0.01
MTMM + VCA						
All SNPs + local (43,569 SNPs)	25.58 (27.54)	67.12 (21.42)	1.11 (0.06)	0.13 (0)	6.06	0.02
*A priori* (3,105 SNPs)	11.53 (9.43)	30.89 (10.87)	16.69 (10.98)	35.33 (8.7)	5.55	0.01

## Discussion

### Mapping polymorphisms responsible for G x E

The main purpose of this study was to investigate the genetic architecture of G x E variation using a population and experimental setting where such variation was massive. Roughly 66% of the variation for flowering time among lines across environments in this study is due to G x E (Table 2), yet a standard GWAS method failed to detect a single significant SNP association. Indeed, even when considering enrichment for *a priori* candidates using less stringent thresholds, there is no trace of G x E associations. The same was true using various summaries of the traits, like the slope of the reaction norm. In contrast, there is ample evidence for polymorphisms that do not interact with the environment (include two that reach genome-wide significance), although this type of variation is only 28% of the phenotypic variation.

The much-discussed “missing heritability” problem in human genetics refers to the fact that individually identifiable (mappable) SNPs do not explain the genetic variation [32]. Although many explanations have been proposed, the simplest one is that the marginal contributions of the underlying variants are too small (due to a combination of allele frequency and effect size) for them to be identified given the statistical power of the study. This explanation is supported by studies that increase power by increasing sample size [33] or that use variance components to estimate the joint contribution of all SNPs rather than trying to identify marginal effects [34].

In the present study, we have no “missing heritability” for common genetic variation, since the SNPs we identified account for almost all of this (22% vs 28%; Tables 2 and 5). However, we do have “missing heritability” for G x E variation, where the identified SNPs explain less than half of the existing variation (31% vs 66%; Tables 2 and 5). Why this difference between G and G x E? The obvious explanation is again power. Under some scenarios, G x E effects are more difficult to detect for purely statistical reasons [7], and it is also possible that the distribution of allele frequencies and/or effect sizes differ. Simulation studies have likewise suggested that substantial genetic risk score-by-environment interactions may exist, although marginal G x E effects are undetectable [35].

The notion that power is involved is supported by the fact that we are able to account for the missing G x E variation fully using variance component methods that estimate the joint contribution of multiple SNPs (Tables 2 and 5). However, these results also demonstrate that the G x E variation is not Fisherian in the sense of being spread out infinitessimally thinly across the genome. Instead, 8 small regions, comprising about 0.5% of the genome, appear to explain almost all the G x E variation (S5 Table). This suggests that G x E variation for flowering is due to a relatively small number of genes harboring a large number of functionally distinct alleles (or haplotypes), *i.e*., allelic rather than genetic heterogeneity. This is consistent with what is known about allelic variation at several flowering time loci [18, 36, 37], and perhaps also with the general observation that different linkage mapping experiments, which are insensitive to allelic heterogeneity, consistently seem to identify the same small number of flowering loci, several of which have not been identified using GWAS [38, 39]. Dissecting these complex regions and haplotypes further will likely require painstaking experimental work, as linkage disequilibrium is typically too extensive for fine-mapping [12, 18].

It should be noted that the extensive allelic heterogeneity for G x E is in contrast to several examples from crops [40, 41]. A possible explanation for this is that domestication and breeding increased the frequency of rare alleles. The pattern in *A. thaliana*, on the other hand, suggests strong local adaptation. There is no obvious correlation between flowering time and geography in our data, but this is not surprising given the strong G x E effects, and the existence of micro-scale climate variation. In order to elucidate the selective forces acting on flowering time variation, field experiments will be required [14, 42].

### Flowering time control in Swedish lines

A secondary purpose of this project was to investigate the genetics of flowering time variation in a local population sample from Sweden. From an *a priori* list of more than hundred flowering time genes, we identified five genes, *FLC*, *SVP*, *VIN3*, *CCT* and *FPA* at an FDR of less than 20% (S5 Table). *FLC*, in particular, clearly has a major effect, in agreement with its role as a major flowering repressor and central player in the vernalization response [43]. Although flowering time is determined by the interaction of huge networks that include the photoperiod, gibberellin, vernalization, temperature, autonomous pathways [44], we found that all identified flowering time genes in our analysis were tightly related to the regulation of *FLC* and *FT* (S7 Fig). Briefly, floral initiation starts immediately by upregulation of *FT* when warm temperature returns after *FLC* is epigenetically silenced by *VIN3* during a cold period [20, 24]. *CCT* and *FPA* suppresses *FLC* in the autonomous pathway [29, 30, 45]. *SVP* has been reported as another flowering regulator that suppresses *FT* independent of *FLC* [46]. It should be noted that *CCT* is closely linked to *FRIGIDA* (*FRI*, distance is 13.97 kbp), a strong up-regulator of *FLC* [47–49] known to harbor, strong allelic heterogeneity and massive haplotype sharing in global samples (over 250 kbp [50, 51]). Although *FRI* is not known to be segregating in the Swedish population, it is clearly possibly that *FRI* alleles could lead to confounding at *CCT* [12]. In addition to known flowering time genes, we also identified one possible novel gene. Although our FDR approach only works for *a priori* candidates, the peak in *FIO1* is clearly significant at the genome-wide level, and the association is currently being confirmed experimentally.

With the exception of *FLC* and *SVP*, none of the genes identified here have previously been shown to be important in natural variation. This demonstrates the advantages of using a local sample for GWAS when working on a trait important in local adaptation, and is in agreement with the G x E results above. Given that allelic heterogeneity can have a major effect on the power of GWAS even within Sweden, it should come as no surprise that flowering time is recalcitrant to GWAS in global samples [12].

## Materials and Methods

### Plant materials and growth conditions

173 Swedish lines and Col-0 were used for experiments (S1 Table). These lines, and all genome information, including SNPs and short indels, are described elsewhere [11].

Seeds were sown on soil and stratified for three days at 4°C in the dark. They were then transferred into a single pot after germination. All plants were grown in MTPS144 Conviron walk-in growth chambers (Winnipeg, MB, Canada) set to long-day conditions (16 h photoperiod) under 10°C or 16°C constant temperatures. Periods from germination to presence of first buds were recorded as flowering time for multi-individuals for each line. Measurements were taken twice a week, until 190 days from germination.

### Statistical analysis

#### Broad sense heritability

The broad-sense heritability (*H* ^2^) was calculated using all individuals as *V*_*G*_/*V*_*P*_, where *V*_*P*_ is the total phenotypic variance and *V*_*G*_ is the genetic variance (estimated from the between-line phenotypic variance).

#### Genome-wide association mapping

For GWAS, the multi-trait mixed model were performed using LIMIX [27] using the model

$$\begin{array}{c} Y∼N(\left[\mu_{10},\mu_{16}\right]⊗1_{n,1}+\left(A⊗x\right)B,\sigma_{g}^{2}\left(C⊗R+\delta Q⊗I\right)), \end{array}$$ (1)

where **Y** is a vector of *n* × *p* phenotypic means (one mean for each of *n* lines in *p* environments), *μ*_10_ and *μ*_16_ are temperature specific mean values, **x** is the vector of genotypes to be tested (SNPs or indels), **A** is a trait design matrix (environment), **B** is the effect size estimate corresponding to **A**, **R** is a genomic relatedness (sample-sample covariance matrix) estimated from SNPs, **C** is the trait-trait covariance matrix, **Q** is a trait-trait noise covariance matrix, and $\sigma_{g}^{2}$ and *δ* are scaling factors. SNPs and indels were analyzed separately in the model and **R** calculated with only SNPs was used for both analyses. Three different tests using likelihood ratio test were carried out [27]:

1. The full model with **A** = **I**_*p*_ tested against a null model *x* = 0. This test identifies “any effect” including environment persistent and specific marker (SNPs or indel) effects between two environments.
2. To identify “interaction effect” (G_SNP_ x E) as environment specific marker effects, the full model was tested against a genetic model as **A** = **1**_1,*p*_.
3. To identify “common SNP effect” as environment persistent marker effects, the genetic model was tested to the null model.

Standard multi-linear regression (MLR) analysis was also conducted using LIMIX function as well as the tests in MTMM. In both MTMM and MLR, Bonferroni-corrected 5% significance thresholds were used. Rare alleles (minor allele frequency less than 10%) were not included in final results and Bonferroni corrections.

#### Variance components analysis (VCA)

VCA was conducted by LIMIX with the model

$$\begin{array}{c} Y=\left[\mu_{10},\mu_{16}\right]⊗1_{n,1}+U_{\text{local}}+U_{\text{global}}+\psi , \end{array}$$ (2)

where **U**_local_ and **U**_global_ are random effects corresponding to local and global relatedness, respectively, and **ψ** is noise. **U**_local_ and **U**_global_ can each be decomposed into an environment-persistent and an environment-specific variant component:

$$\begin{array}{c} U_{\text{local}}∼N(0,(c_{p}^{2}1_{p,p}+[\begin{array}{cc} c_{1}^{2} & 0\\ 0 & c_{2}^{2} \end{array}])⊗R_{\text{local}}); \end{array}$$ (3)

$$\begin{array}{c} U_{\text{global}}∼N(0,(t_{p}^{2}1_{p,p}+[\begin{array}{cc} t_{1}^{2} & 0\\ 0 & t_{2}^{2} \end{array}])⊗R_{\text{global}}); \end{array}$$ (4)

$$\begin{array}{c} \psi ∼N(0,([\begin{array}{cc} \sigma_{1}^{2} & \sigma_{1,2}\\ \sigma_{1,2} & \sigma_{2}^{2} \end{array}])⊗I). \end{array}$$ (5)

Here **R**_local_ and **R**_global_ are sample-sample covariance matrices that estimate genetic relatedness (kinship) based on local and global SNPs, respectively. The local region of a gene was defined as the gene body plus 15 kbp from the 5’ and 3’ UTR, respectively, and global was defined as the rest of the genome. The parameters $c_{p}^{2}$ and $t_{p}^{2}$ are environment-persistent variances and covariances for the local and global genetic terms, and $\sigma_{1}^{2}$, $\sigma_{2}^{2}$, *σ*_1,2_ are the noise covariance parameters. To evaluate the “full local” (including environment persistent and specific effect), “common local” (environment persistent effect) and “G_local_ x E” (environment specific effect) effects, three different tests were carried out and log likelihood-ratio was calculated:

1. A “full local effect” was tested by comparison of a full model, including local and global effects and their interactions with E, with a null model that does not include any local effect (**U**_local_).
2. A “common local effect” was tested by comparison of a local model that does not include an interaction effect between the local effect and E (as $c_{1}^{2}$, $c_{2}^{2}=0$) with the null model.
3. An “interaction (G_local_ x E) effect” was tested by comparison of the full model with the local model.

The null model was also used to determine genetic (global), environmental and G x E effects on flowering time variations in Table 2.

#### Quantile-quantile plots

Quantile-quantile plots were constructed by the rank of significance of all flowering time genes and the corresponding non-flowering time genes. For GWAS, the most significant *p*-value within 15 kbp from a gene was assigned for significance of the gene. First, genes in each flowering and non-flowering time gene lists were ranked according to significance of these genes from smallest to largest, and the ranks were scaled by number of genes in the list. We assumed (as a null hypothesis) that a distribution of significances of genes in both lists are same, and genes that have a same rank after scaling will have same significance. To help interpretation of the plots, 95% confidence interval was calculated (shaded grey in all quantile-quantile plots). For this, we conducted random sampling (1000 times) that maintained the chromosomal order of all observations but shuffled the relative positions of the two variables (for details see [52]). Random distributions were generated point by point and the 2.5th and 97.5th percentiles of each point were calculated from the distribution.

#### Enrichment test and bounding the FDR

Observed enrichments were assessed to optimize the threshold of MTMM and VCA according to the method of Atwell *et al*. [12]. Briefly, if we assume that all non-candidate genes are false, then we can estimate the fraction of true positives and false positives among the *a priori* candidates. We estimated the enrichment as *X*/*Y* and the FDR as

$$\begin{array}{c} {\text{FDR}}_{\text{upper}\%}=1-\left(X-Y\right)/X=Y/X, \end{array}$$ (6)

where *Y* is the fraction of non *a priori* genes (S3 Table) that are significant, and *X* is the fraction of *a priori* genes that are significant. 113 functionally confirmed flowering time genes were used for *a priori* list. For GWAS, the most significant *p*-value within 15 kbp of a gene was assigned as the significance of that gene.

## Supporting Information

S1 Fig Geographic origin of Swedish lines that were used in this study. Red points show the places where accessions were collected.
(PDF)

S2 Fig Quantile-quantile plots of GWAS *p*-values. Top row, MTMM; bottom row, MLR. Colored lines show results after removing 30 kbp regions containing a peak more significant than the value listed.
(PDF)

S3 Fig Amino acid sequences of *FIO1*. (PDF)

S4 Fig Manhattan plots of GWAS results for flowering time at 10°C and 16°C using MLR. From top to bottom, results for full SNP test, common SNP effects, and G_SNP_ x E effects.
(PDF)

S5 Fig Correlation between significance of genes by VCA and MTMM. Blue points indicate *a priori* flowering time genes and green points indicate the other genes.
(PDF)

S6 Fig Distribution of local variance component by permutation test. Full local variance component and the log-likelihood ratios for all local (top) and *a priori* list (bottom). Orange lines show actual estimated values in Table 5. Random sampling (1000 times) was conducted with maintaining the chromosomal order of all observations but shuffling the relative positions of the two variables.
(PDF)

S7 Fig A flowering time regulation network that is predicted by genome-wide screening in this study. Boxes indicate genes that are involved in the network (environment-persistent effects in orange; environment-specific effects in blue; both effects in purple). Arrows indicate positive effects (upregulation) and T arrows indicate negative effects (suppression). Black arrows show experimentally confirmed regulations in previous studies. Dot arrows show hypothesized associations.
(PDF)

S1 Table Accession used with average phenotypes. (PDF)

S2 Table Raw flowering time data (incl. replicates). (CSV)

S3 Table List of flowering time genes known *a priori*. (PDF)

S4 Table All associations significant at the 10^−5^ level. (PDF)

S5 Table Summary of associations in MTMM and VCA. (PDF)
