# Supplementary figures and images for: "Missing" G x E Variation Controls Flowering Time in Arabidopsis thaliana
Source: PLoS Genet. 2015 Oct 16;11(10):e1005597. doi: 10.1371/journal.pgen.1005597 (PMC4608753; doi:10.1371/journal.pgen.1005597)

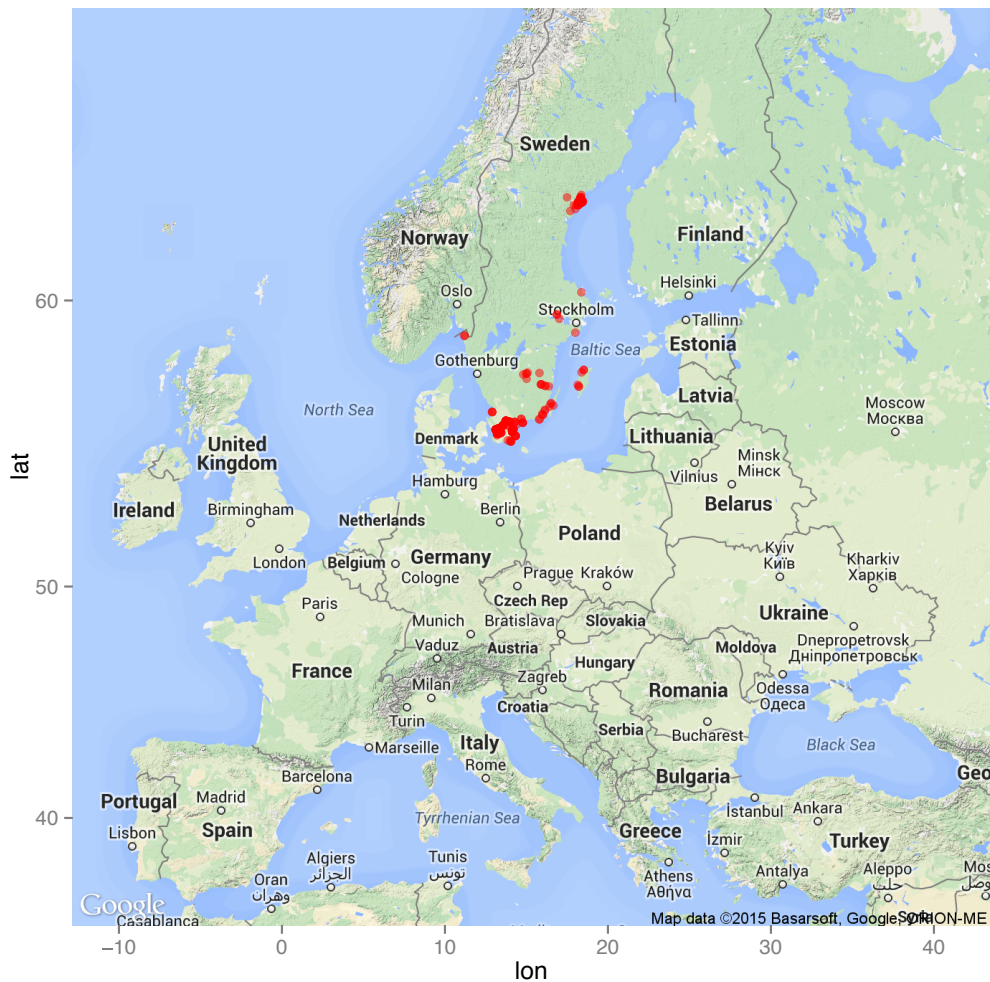

Supplement: S1 Fig — Red points show the places where accessions were collected. (PDF) [file pgen.1005597.s001.pdf]

# MTMM

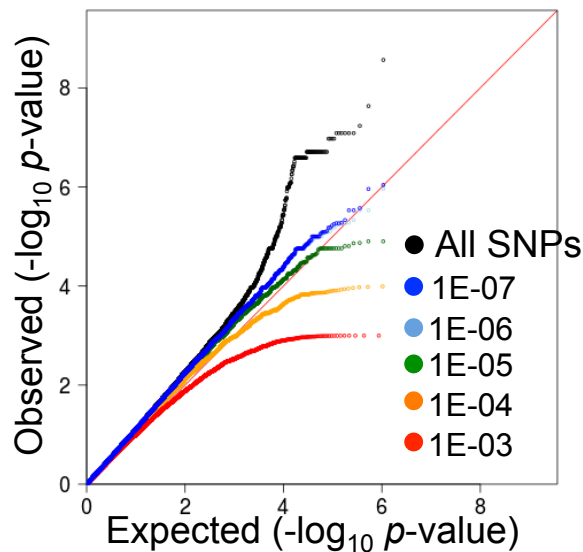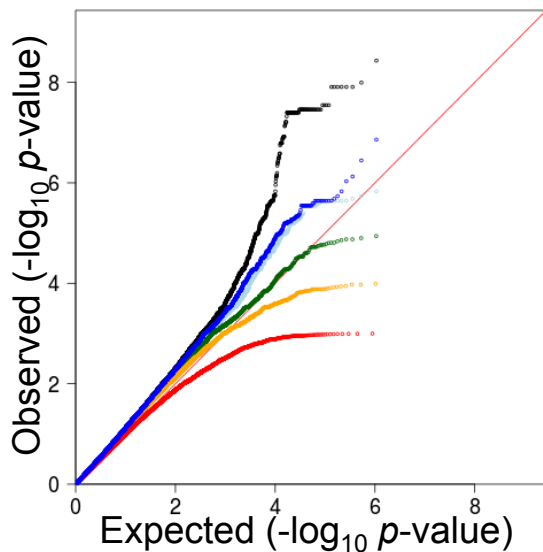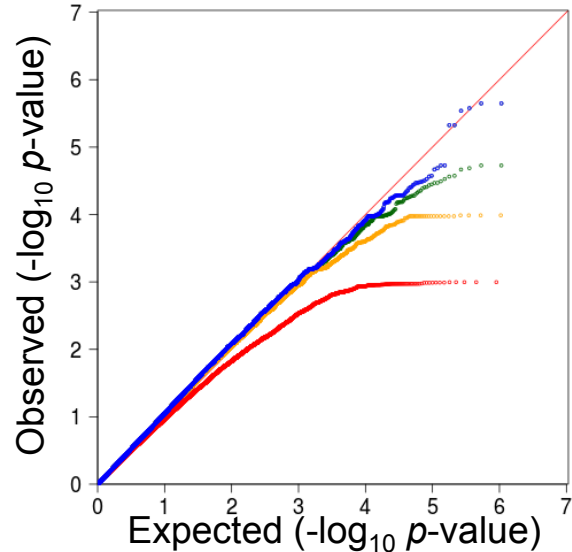

# MLR

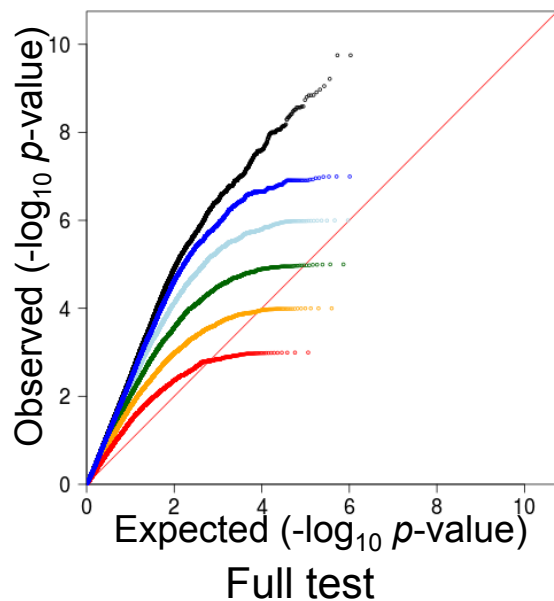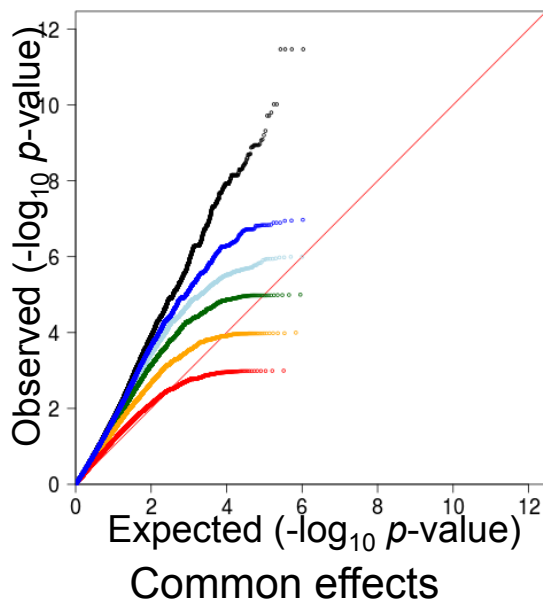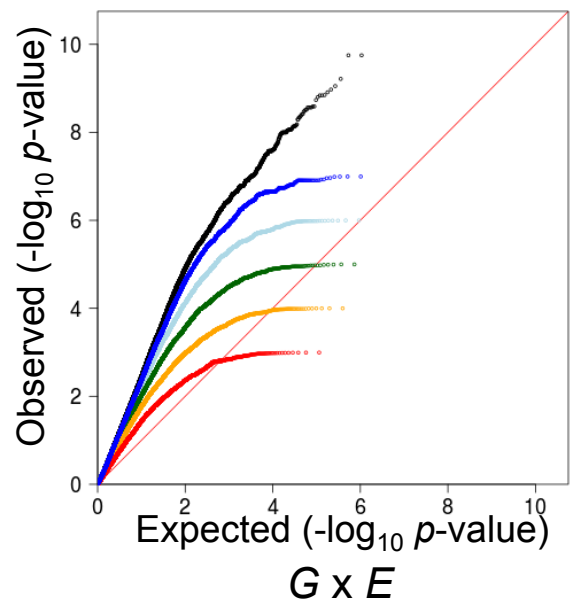

Supplement: S2 Fig — Top row, MTMM; bottom row, MLR. Colored lines show results after removing 30 kbp regions containing a peak more significant than the value listed. (PDF) [file pgen.1005597.s002.pdf]

Haplotype 1  
Haplotype 2  
Haplotype 3

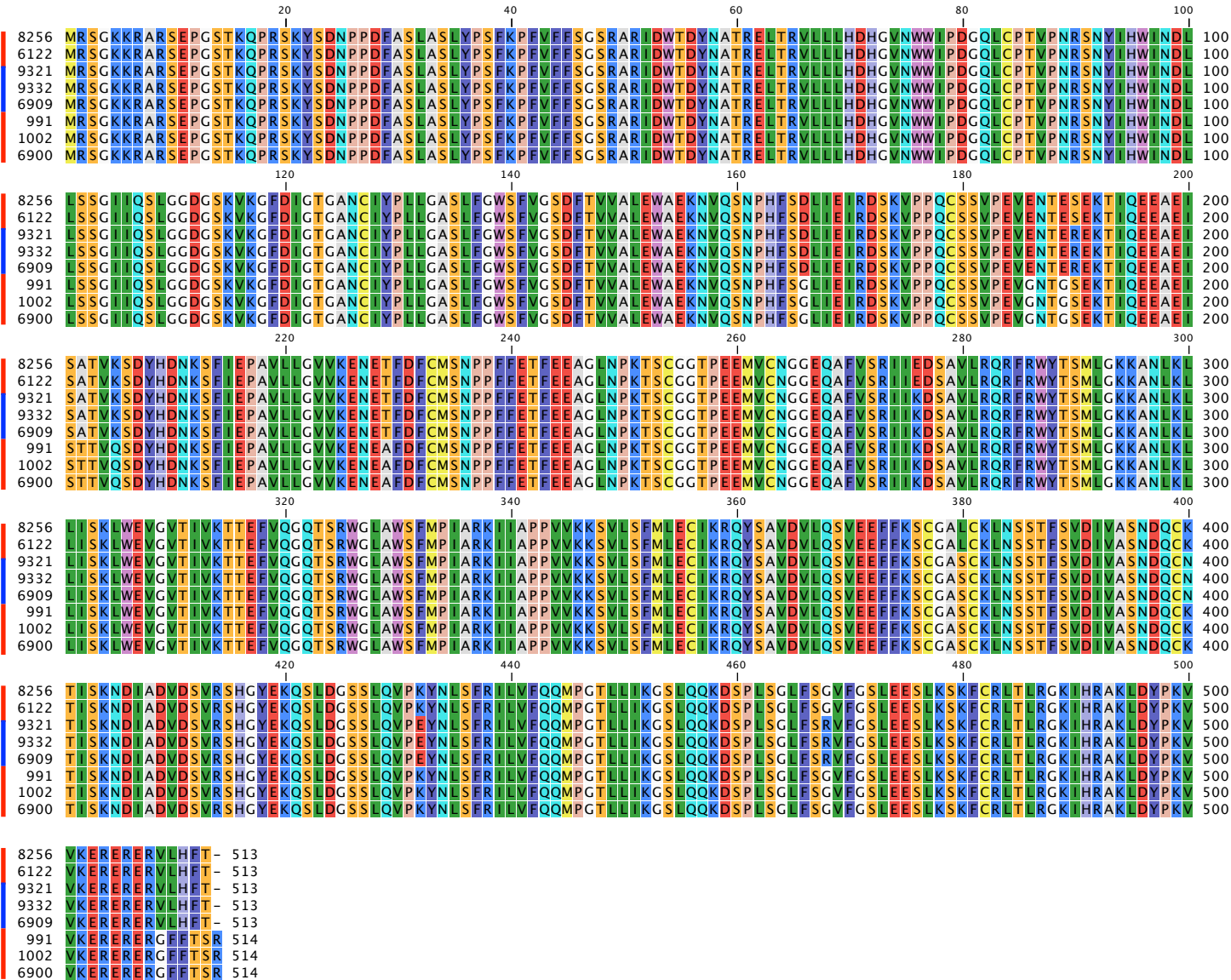

Supplement: S3 Fig — (PDF) [file pgen.1005597.s003.pdf]

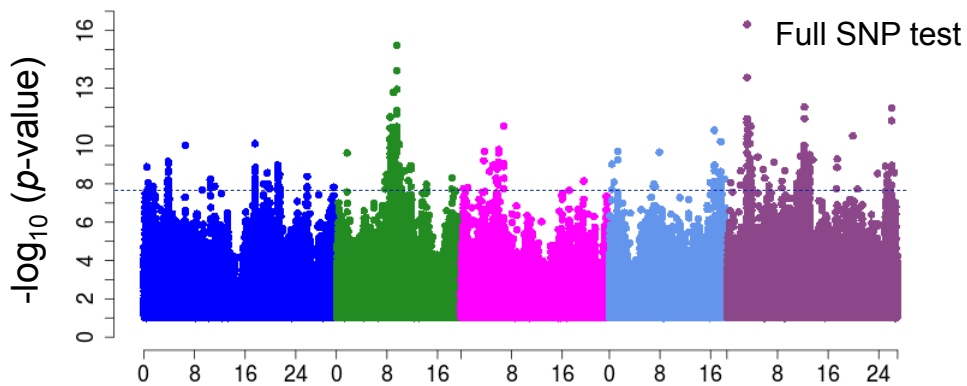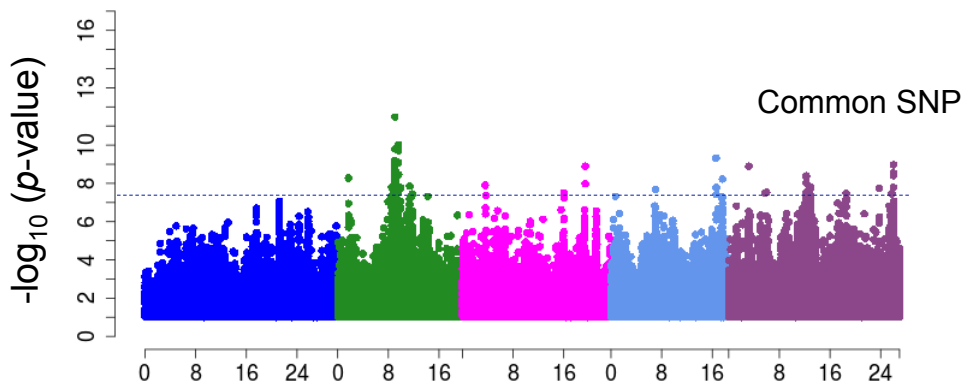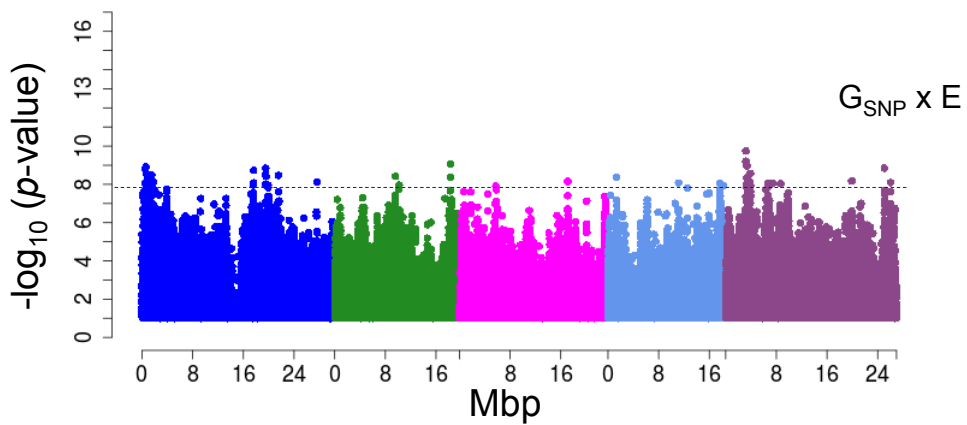

Supplement: S4 Fig — From top to bottom, results for full SNP test, common SNP effects, and GSNP x E effects. (PDF) [file pgen.1005597.s004.pdf]

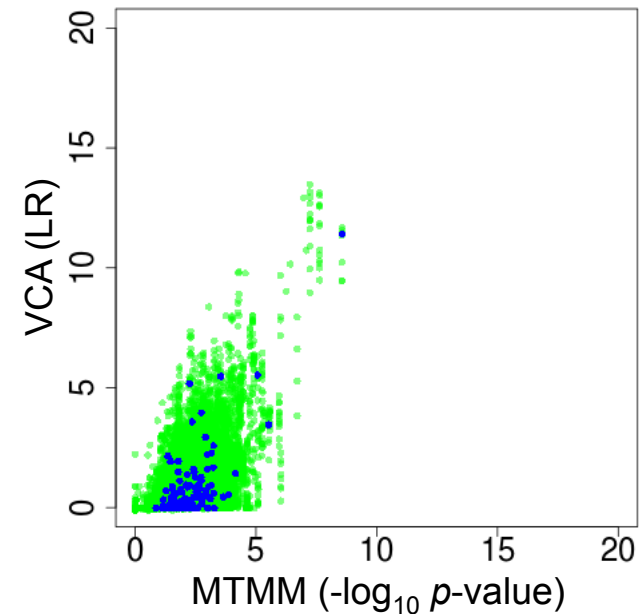

Full test

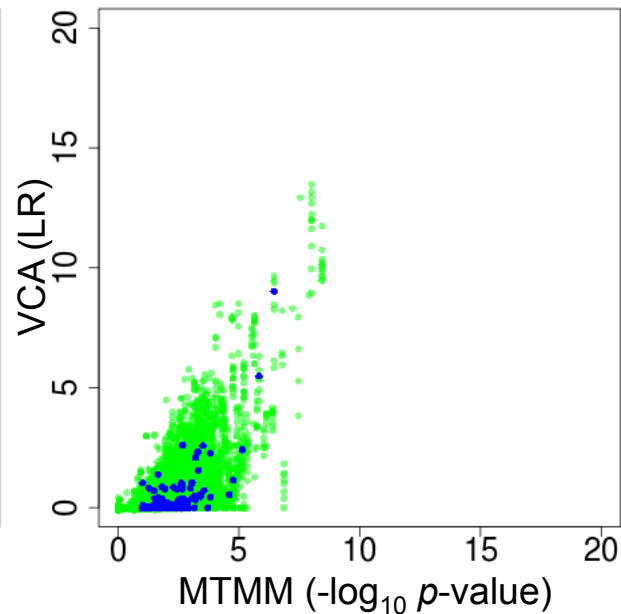

Common effect

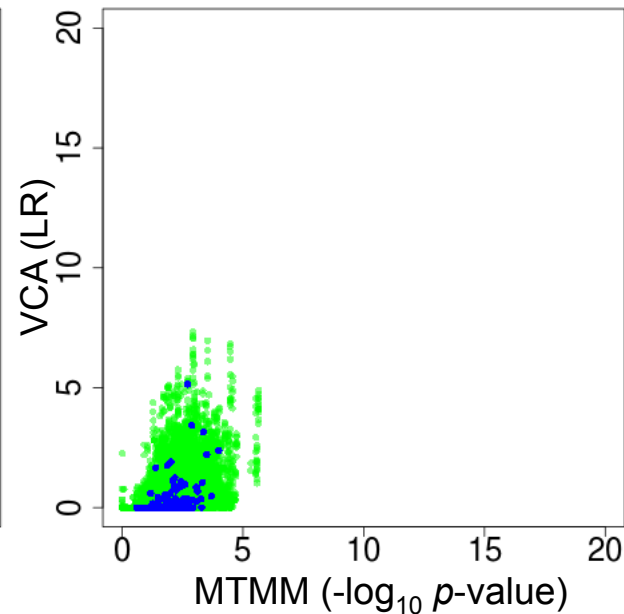

G x E

Supplement: S5 Fig — Blue points indicate a priori flowering time genes and green points indicate the other genes. (PDF) [file pgen.1005597.s005.pdf]

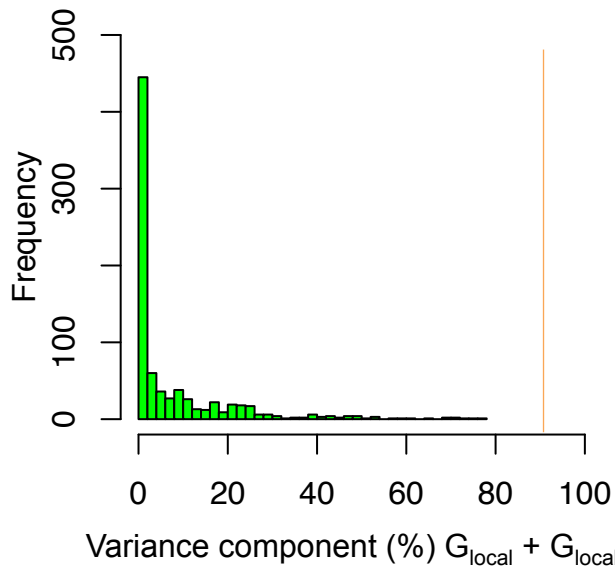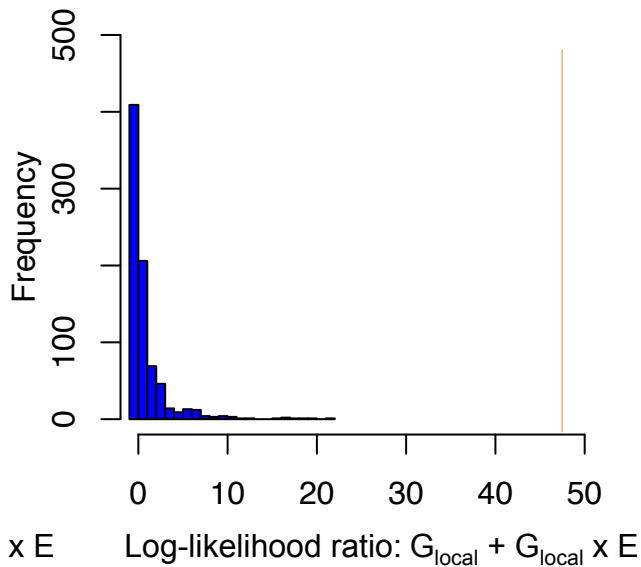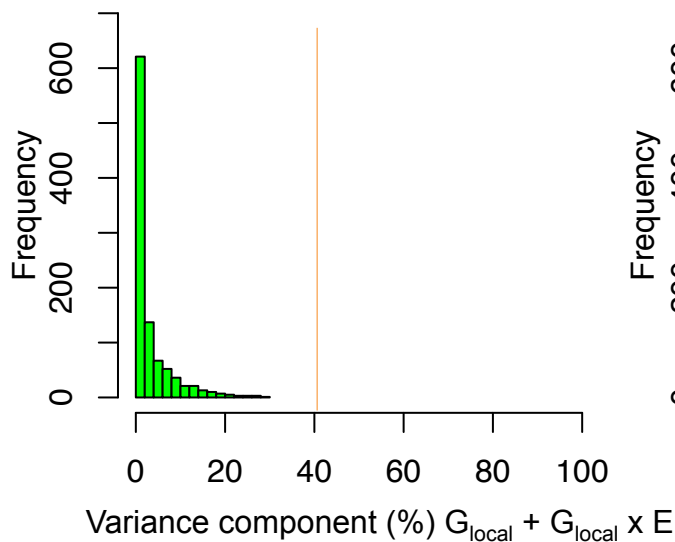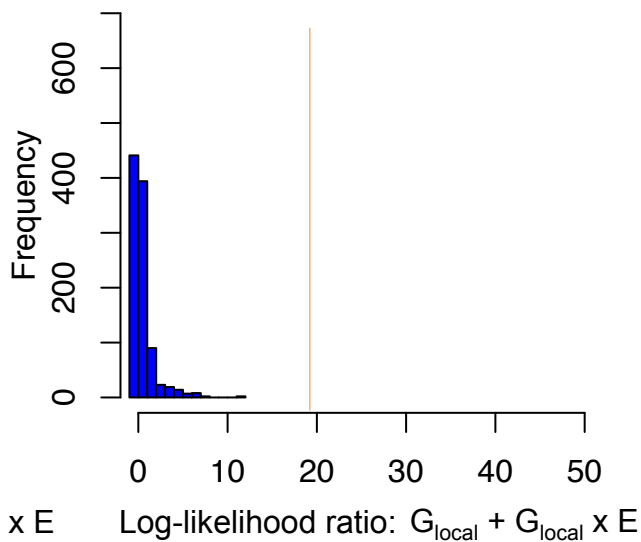

Supplement: S6 Fig — Full local variance component and the log-likelihood ratios for all local (top) and a priori list (bottom). Orange lines show actual estimated values in Table 5. Random sampling (1000 times) was conducted with maintaining the chromosomal order of all observations but shuffling the relative positions of the two variables. (PDF) [file pgen.1005597.s006.pdf]

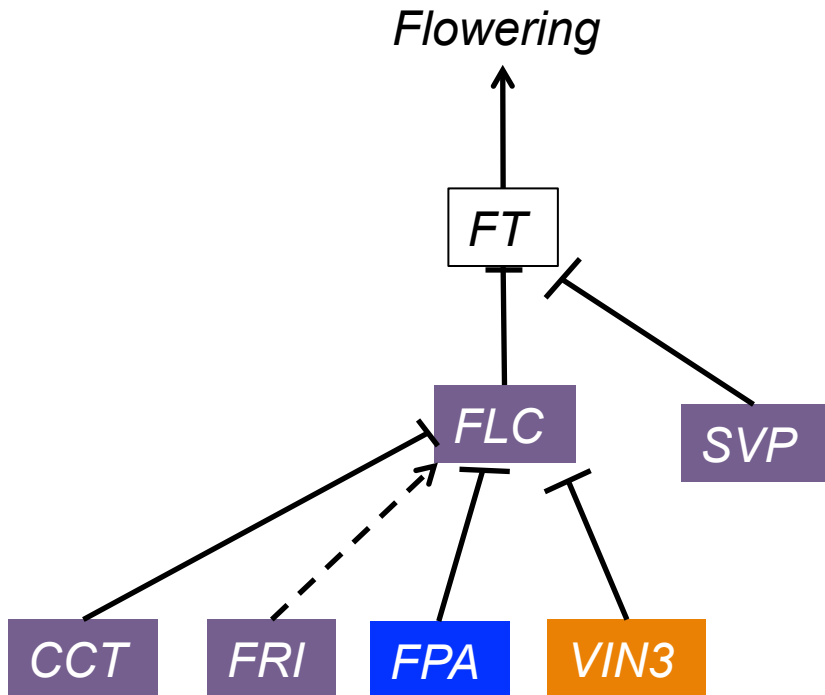

Supplement: S7 Fig — Boxes indicate genes that are involved in the network (environment-persistent effects in orange; environment-specific effects in blue; both effects in purple). Arrows indicate positive effects (upregulation) and T arrows indicate negative effects (suppression). Black arrows show experimentally confirmed regulations in previous studies. Dot arrows show hypothesized associations. (PDF) [file pgen.1005597.s007.pdf]
