# Supplementary material for: "Missing" G x E Variation Controls Flowering Time in Arabidopsis thaliana
Source: PLoS Genet. 2015 Oct 16;11(10):e1005597. doi: 10.1371/journal.pgen.1005597 (PMC4608753; doi:10.1371/journal.pgen.1005597)
Supplement: S1 Table — (PDF) [file pgen.1005597.s008.pdf]

Table S1. Accession used with average phenotypes

Ecotype ID	Line name	Latitude	Longitude	10°C	16°C	*p*-val	*q*-val	Ratio	Group*
991	Ale-Stenar-41-1	55.3833	14.05	97	176	0.00014	0.00018	1.81	D
992	Ale-Stenar-44-4	55.3833	14.05	100	160	0.1	0.043	1.6	N
1006	Ale-Stenar-77-31	55.3833	14.05	100	147	0.16	0.062	1.47	N
1061	Brösarp-11-135	55.7167	14.1333	96	88	0.0018	0.0015	0.92	A
1062	Brösarp-15-138	55.7167	14.1333	82	67	0.084	0.0345	0.82	N
1063	Brösarp-21-140	55.7167	14.1333	75	55	1.18E-05	2.56E-05	0.73	A
1070	Brösarp-45-153	55.7167	14.1333	80	142	0.12	0.048	1.78	N
1074	Brösarp-61-162	55.7167	14.1333	91	129	0.061	0.0268	1.41	N
1137	Gårdby-22-213	56.6167	16.65	89	155	0.00033	0.00037	1.74	D
1166	Aledal-14-73	56.7	16.5167	97	85	0.0023	0.0019	0.87	A
1254	Tos-82-387	59.4333	17.0167	89	103	0.44	0.14	1.16	N
1313	Ängsö-59-422	59.5667	16.8667	79	101	0.036	0.018	1.28	N
1317	Ängsö-74-430	59.5667	16.8667	91	80	8.89E-05	0.00013	0.88	A
1435	Röd-17-319	62.8	18.2	61	47	0.018	0.010	0.78	N
1552	Sku-30	63.0833	18.3667	85	119	0.14	0.054	1.4	N
5832	App1-16	56.3333	15.9667	87	66	0.00114	0.0014	0.76	A
5856	Dör-10	63.0167	17.4914	119	107	0.45	0.14	0.9	N
5867	Dra2-1	55.76	14.12	123	190	9.6E-05	0.00014	1.55	D
6010	Eden-5	62.877	18.177	104	112	0.97	0.27	1.07	N
6011	Eden-6	62.877	18.177	85	95	0.60	0.18	1.12	N
6012	Eden-7	62.877	18.177	95	126	0.059	0.026	1.33	N
6013	Eden-9	62.877	18.177	101	101	0.29	0.096	1	N
6016	Eds-1	62.9	18.4	75	89	0.23	0.079	1.19	N
6019	Fjä1-2	56.06	14.29	103	102	0.133	0.051	0.99	N
6021	Fjä2-4	56.06	14.29	102	86	3.94E-05	7.30E-05	0.84	A
6023	Fly2-1	55.7509	13.3712	94	77	0.025	0.014	0.82	N
6030	Grön-5	62.806	18.1896	105	153	0.080	0.033	1.46	N
6034	Hov1-7	56.1	13.74	92	87	0.044	0.021	0.95	N
6036	Hov3-2	56.1	13.74	83	68	0.039	0.0194	0.82	N
6038	Hov3-5	56.1	13.74	86	70	0.035	0.018	0.82	N
6039	Hovdala-2	56.1	13.74	66	46	4.96E-05	8.56E-05	0.7	A
6040	Kni-1	55.66	13.4	71	75	0.61	0.18	1.06	N
6041	Lis-3	56.0328	14.775	73	48	3.13E-07	1.25E-06	0.66	A
6043	Löv-1	62.801	18.079	90	190	6.32E-06	1.64E-05	2.11	D
6046	Löv-5	62.801	18.079	93	190	5.93E-08	4.40E-07	2.04	D
6064	Nyl-2	62.9513	18.2763	96	138	0.25	0.085	1.44	N
6069	Nyl-7	62.9513	18.2763	111	107	0.077	0.032	0.96	N
6070	Omn-1	62.9308	18.3448	96	125	0.014	0.0084	1.3	D
6071	Omn-5	62.9308	18.3448	109	125	0.0081	0.0054	1.15	D
6073	ÖMö1-7	56.1481	15.8155	107	89	5.07E-06	1.38E-05	0.83	A
6074	Ör-1	56.4573	16.1408	91	83	0.076	0.032	0.91	N
6076	Rev-2	55.6942	13.4504	115	148	0.00026	0.00031	1.29	D
6077	Rev-3	55.6942	13.4504	105	107	0.25	0.084	1.02	N
6085	Sparta-1	55.7097	13.2145	125	190	8.65E-10	4.11E-08	1.52	D
6086	Sr:3	58.9	11.2	92	83	1.97E-07	1.02E-06	0.9	A
6088	Stu1-1	56.4666	16.1284	93	140	0.051	0.023	1.51	N
6090	T1000	55.6525	13.2197	92	90	0.050	0.023	0.98	N
6092	T1020	55.6514	13.2233	104	79	0.0032	0.0024	0.76	A

Continued

Ecotype ID	Line name	Latitude	Longitude	10°C	16°C	p-val	q-val	Ratio	Group
6096	T1060	55.6472	13.2225	98	165	0.0067	0.0046	1.68	D
6098	T1080	55.6561	13.2178	85	109	0.87	0.248	1.28	N
6099	T1090	55.6575	13.2386	111	114	0.68	0.20	1.03	N
6102	T1130	55.6	13.2	118	190	4.17E-07	1.48E-06	1.61	D
6104	T1160	55.7	13.2	101	115	0.70	0.21	1.14	N
6105	T450	55.7967	13.1211	103	118	0.18	0.066	1.15	N
6106	T460	55.7931	13.1186	105	121	0.20	0.069	1.15	N
6107	T470	55.7942	13.1222	113	151	0.059	0.026	1.34	N
6108	T480	55.7989	13.1206	74	59	0.012	0.0075	0.8	A
6109	T510	55.7936	13.1233	118	135	0.54	0.17	1.14	N
6112	T540	55.7967	13.1044	97	74	0.00011	0.00016	0.76	A
6114	T570	55.8097	13.1342	104	95	0.0079	0.0053	0.92	A
6118	T610	55.7	13.2	103	105	0.034	0.018	1.02	N
6119	T620	55.7	13.2	113	131	0.18	0.065	1.16	N
6122	T670	55.8364	13.3075	96	104	0.81	0.23	1.08	N
6123	T680	55.8369	13.3033	106	85	0.00012	0.00016	0.8	A
6124	T690	55.8378	13.3092	125	113	0.0064	0.0045	0.9	A
6125	T710	55.8403	13.3106	108	117	0.84	0.24	1.08	N
6126	T720	55.8411	13.3047	119.7	107	0.060	0.026	0.89	N
6127	T730	55.8428	13.3058	106	172	0.00089	0.00089	1.63	D
6132	T790	55.8386	13.3186	103	84	0.0025	0.0020	0.81	A
6136	T840	55.9336	13.5519	86	86	0.036	0.018	1	N
6140	T880	55.9392	13.5539	86	77	0.044	0.021	0.9	N
6141	T890	55.9414	13.5542	91	158	0.00061	0.00063	1.74	D
6142	T900	55.9428	13.5558	90	101	0.37	0.12	1.12	N
6147	T950	55.9425	13.5675	106	165	0.023	0.013	1.56	N
6149	T970	55.9281	13.5481	89	110	0.12	0.046	1.24	N
6150	T980	55.9261	13.5319	84	84	0.25	0.084	1.01	N
6151	T990	55.6528	13.2244	108	102	0.23	0.080	0.94	N
6169	TÅD 01	62.8714	18.3447	91.5	120	0.0011	0.00097	1.31	D
6171	TÅD 03	62.8717	18.3444	104	128	0.30	0.10	1.23	N
6172	TÅD 04	62.8717	18.3436	80	100	0.20	0.070	1.25	N
6173	TÅD 05	62.8717	18.3419	90	78	0.0015	0.0013	0.87	A
6180	TÄL 07	62.6322	17.6906	59	33	0.00013	0.00017	0.56	A
6184	TBÖ 01	62.8892	18.4522	85	95	0.73	0.21	1.11	N
6188	TDr-1	55.7683	14.1386	59	41	1.71E-06	5.21E-06	0.69	A
6189	TDr-2	55.7686	14.1383	73	41	8.08E-08	5.24E-07	0.56	A
6191	TDr-4	55.7689	14.1375	68	49	4.56E-05	8.14E-05	0.72	A
6192	TDr-5	55.7692	14.1369	75.8	46	4.28E-07	1.48E-06	0.61	A
6193	TDr-7	55.7694	14.1347	71	45	8.31E-05	0.00013	0.63	A
6194	TDr-8	55.7706	14.1342	109	83	0.0035	0.0026	0.76	A
6195	TDr-9	55.7708	14.1342	68	54	0.00096	0.00094	0.79	A
6198	TDr-13	55.7708	14.1331	79	62	0.010	0.0066	0.79	A
6202	TDr-17	55.7717	14.1206	115	129	0.31	0.10	1.12	N
6203	TDr-18	55.7714	14.1208	103	145	0.066	0.028	1.41	N
6209	TEDEN 02	62.8836	18.1842	90	112	0.11	0.044	1.24	N
6210	TEDEN 03	62.8839	18.1836	89	106	0.13	0.050	1.19	N
6216	TFÄ 06	63.0167	18.3283	118	190	2.62E-07	1.19E-06	1.62	D
6218	TFÄ 08	63.0172	18.3283	106	183	0.0032	0.0024	1.73	D

Continued

Ecotype ID	Line name	Latitude	Longitude	10°C	16°C	*p*-val	*q*-val	Ratio	Group
6221	TGR 02	62.806	18.1896	105	170	0.10	0.040	1.62	N
6231	TNY 04	62.96	18.2844	110	190	5.32E-05	8.90E-05	1.73	D
6238	TOM 04	62.9619	18.35	99	120	0.016	0.0097	1.21	D
6240	TOM 06	62.9622	18.35	91	146	0.011	0.0067	1.6	D
6242	Tomegap-2	55.7	13.2	94	84	0.23	0.081	0.9	N
6243	Tottarp-2	55.95	13.85	56	41	0.0011	0.00010	0.73	A
6244	TRÄ 01	62.9169	18.4728	108	101	0.49	0.15	0.94	N
6258	TV-10	55.5796	14.3336	107	131	0.68	0.20	1.22	N
6276	TV-30	55.5796	14.3336	109	151	0.19	0.068	1.39	N
6413	Ull3-4	56.06	13.97	113	121	1	0.27	1.07	N
6909	Col-0	38.3	-92.3	51	42	0.048	0.022	0.82	N
6964	Spr1-2	56.3	16	93	127	0.14	0.052	1.37	N
6973	Ull2-3	56.0648	13.9707	53	40	1.59E-09	4.11E-08	0.75	A
6974	Ull2-5	56.0648	13.9707	103	137	0.023	0.013	1.33	N
7516	Vår2-1	55.58	14.334	100	131	0.044	0.021	1.31	N
7517	Vår2-6	55.58	14.334	107	143	0.040	0.020	1.34	N
7519	ÖMö2-3	56.1509	15.7735	76	84	0.62	0.19	1.11	N
8222	Lis-2	56.0328	14.775	90	77	0.0043	0.0031	0.86	A
8227	THÖ 03	62.7989	17.9103	113	155	0.019	0.010	1.37	N
8230	Algutsrum	56.68	16.5	97	119	0.47	0.15	1.23	N
8231	Bröl-6	56.3	16	91	109	0.17	0.062	1.2	N
8240	Kulturen-1	55.705	13.196	93	100	0.99	0.27	1.08	N
8241	Liarum	55.9473	13.821	73	53	7.35E-06	1.66E-05	0.73	A
8247	San-2	56.07	13.74	87	77	0.015	0.0089	0.89	A
8249	Vimmerby	57.7	15.8	81	68	0.00051	0.00054	0.84	A
8256	Bå1-2	56.4	12.9	61	41	0.0062	0.0045	0.67	A
8258	Bå4-1	56.4	12.9	73	50	0.00030	0.00035	0.68	A
8259	Bå5-1	56.4	12.9	73	48	0.0030	0.0023	0.66	A
8283	Dra3-1	55.76	14.12	71	157	0.01043	0.0067	2.21	D
8306	Hov4-1	56.1	13.74	96	107	0.90	0.25	1.11	N
8307	Hovdala-6	56.1	13.74	71	52	1.77E-08	3.06E-07	0.73	A
8326	Lis-1	56.0328	14.775	67	49	2.64E-08	3.42E-07	0.73	A
8334	Lu-1	55.71	13.2	64	46	0.0014	0.0012	0.72	A
8335	Lund	55.71	13.2	104	110	0.00011	0.00016	1.06	D
8351	Ost-0	60.25	18.37	78	139	0.082	0.033	1.78	N
8369	Rev-1	55.6942	13.4504	76	64	3.97E-06	1.14E-05	0.84	A
8376	Sanna-2	62.69	18	84	156	0.18	0.065	1.86	N
8386	Sr:5	58.9	11.2	69	43	2.99E-05	5.75E-05	0.62	A
8387	St-0	59	18	52	45	0.067	0.028	0.87	N
8422	Fjå1-1	56.06	14.29	106	121	0.80	0.23	1.14	N
8426	Ull1-1	56.06	13.97	49	32	5.95E-08	4.40E-07	0.65	A
8427	Ull2-13	56.0648	13.9707	64	40	1.36E-07	7.86E-07	0.63	A
9323	Ådal 3	62.8622	18.336	100.3	116	0.578829732	0.176506451	1.16	N
9332	Bar 1	62.8698	18.381	90	121	0.047	0.022	1.34	N
9336	Bön 1	62.8794	18.4473	85	53	0.000407286	0.00044	0.63	A
9339	Böt 1	57.7133	15.0689	97	125	0.044	0.021158495	1.29	N
9343	Dju-1	57.3089	18.1512	91	119	0.077	0.032	1.31	N
9353	Död 3	57.2608	16.3675	106	190	1.23E-06	3.98E-06	1.79	D
9363	EdJ 2	62.9147	18.4045	120	151	0.44	0.14	1.26	N
9369	EkS 2	57.6781	14.9986	80	69	0.014	0.0086	0.86	A

Continued

Ecotype ID	Line name	Latitude	Longitude	10°C	16°C	*p*-val	*q*-val	Ratio	Group
9370	EkS 3	57.6781	14.9986	78	64	0.056	0.025	0.82	N
9371	FöL 1	63.016	18.3175	106	190	0.00036	0.00040	1.79	D
9380	FlyA 3	55.7488	13.3742	99	105	0.94	0.26	1.07	N
9381	Fri 1	55.8106	14.2091	96	83	1.87E-05	3.84E-05	0.86	A
9382	Fri 2	55.8106	14.2091	99	151	0.0028	0.0022	1.52	D
9383	Fri 3	55.8106	14.2091	96	104	0.92	0.26	1.09	N
9386	Grön 12	62.806	18.1896	117	115	0.053	0.024	0.98	N
9390	Hadd-1	57.3263	15.8979	70	42	5.43E-08	4.40E-07	0.6	A
9391	Hadd-2	57.3263	15.8979	73	47	0.00016	0.00020	0.64	A
9392	Hadd-3	57.3263	15.8979	67	45	6.09E-05	9.86E-05	0.67	A
9394	Hag-2	56.5804	16.4063	104	142	0.15	0.055	1.37	N
9395	Hal-1	57.5089	15.0105	98	97	0.024	0.013	0.99	N
9399	Hamm-1	55.4234	13.9905	102	119	0.23	0.081	1.17	N
9412	Kor 3	57.2746	16.1494	87	61	0.029	0.016	0.71	N
9413	Kor 4	57.2746	16.1494	76	52	2.75E-07	1.19E-06	0.68	A
9416	Kru-3	57.7215	18.3837	115	190	0.001	0.00010	1.65	D
9421	Lan 1	55.9745	14.3997	105	112	0.93	0.26	1.07	N
9427	Näs 2	62.8815	18.4055	91	132	0.026	0.014	1.45	N
9434	Öde 2	62.8959	18.3659	108	161	0.020	0.011	1.49	N
9436	Puk-1	56.1633	14.6806	99	119	0.00029	0.00034	1.2	D
9437	Puk-2	56.1633	14.6806	77	51	7.76E-05	0.00012	0.66	A
9451	Spro 2	57.2545	18.2109	111	179	1.93E-05	3.84E-05	1.61	D
9452	Spro 3	57.2545	18.2109	106	94	0.036	0.018	0.89	N
9453	Ste 2	57.8009	18.5162	98	130	0.47	0.15	1.33	N
9455	Ste 4	57.8009	18.5162	100	116	0.66	0.20	1.16	N
9470	Tur-4	57.6511	14.8043	91	107	0.28	0.094	1.18	N
9481	Yst-1	55.4242	13.8484	107	137	0.00077	0.00078	1.28	D

* A: Accelerated flowering, D: Decelerated flowering, N: No response
