# Supplementary material for: "Missing" G x E Variation Controls Flowering Time in Arabidopsis thaliana
Source: PLoS Genet. 2015 Oct 16;11(10):e1005597. doi: 10.1371/journal.pgen.1005597 (PMC4608753; doi:10.1371/journal.pgen.1005597)
Supplement: S3 Table — (PDF) [file pgen.1005597.s010.pdf]

**Table S3. List of flowering time genes known *a priori***

| AGI code | Gene name | Pathway | Reference |
|---|---|---|---|
| AT1G01060 | LHY | | [1, 2, 3, 4] |
| AT1G04400 | CRY1 | Photoperiod | [1] |
| AT1G05830 | ATX2 | Vernalization | [5] |
| AT1G08970 | NF-YC | Photoperiod | [6] |
| AT1G09030 | NF-YB | Photoperiod | [6] |
| AT1G09570 | PHYA | Photoperiod | [4, 1] |
| AT1G12610 | DDF1 | | [7] |
| AT1G14920 | GAI | GA | [1] |
| AT1G17760 | CSTF77 | | [1] |
| AT1G22770 | GI | | [1] |
| AT1G24260 | SEPALLATA3 | | [6] |
| AT1G25560 | TEM1 | | [1] |
| AT1G26310 | CAL | | [6] |
| AT1G30970 | SUF4 | | [8] |
| AT1G43850 | SEU | | [6] |
| AT1G53090 | SPA4 | Photoperiod | [1] |
| AT1G53160 | SPL4 | | [2] |
| AT1G63030 | DDF2 | | [7] |
| AT1G65480 | FT | Integrator | [1, 2] |
| AT1G68050 | FKF1 | Photoperiod | [1] |
| AT1G68840 | TEM2 | | [1] |
| AT1G69120 | AP1 | | [1, 2] |
| AT1G71800 | CSTF64 | | [1] |
| AT1G77080 | FLM | | [1] |
| AT1G78580 | TPS1 | | [1] |
| AT2G01570 | RGA | GA | [1] |
| AT2G17770 | FDP | | [9] |
| AT2G18790 | PHYB | Photoperiod | [1, 4] |
| AT2G19425 | MIR156G | Age regulated | [1] |
| AT2G19520 | FVE | Autonomous | [1] |
| AT2G22540 | SVP | Ambient temperature | [1] |
| AT2G23380 | CLF | | [1] |
| AT2G25095 | MIR156A | Age regulated | [1] |
| AT2G25930 | ELF3 | | [3, 10, 4] |
| AT2G28056 | MIR172A | Photoperiod | [1] |
| AT2G28550 | TOE1 | | [1, 2] |
| AT2G31650 | ATX1 | Vernalization | [5] |
| AT2G32950 | COP1 | Photoperiod | [1] |
| AT2G33810 | SPL3 | | [6, 2] |
| AT2G33835 | FES1 | | [11] |
| AT2G39250 | SNZ | Photoperiod | [1] |
| AT2G39810 | HOS1 | Autonomous | [12] |
| AT2G40080 | ELF4 | | [10, 4] |
| AT2G42200 | SPL9 | GA | [6, 2] |
| AT2G43010 | PIF4 | | [2] |
| AT2G43410 | FPA | Autonomous | [1] |
| AT2G45660 | SOC1 | Integrator | [1] |
| AT2G46340 | SPA1 | Photoperiod | [1] |

Continued

| AGI code | Gene name | Pathway | Reference |
|---|---|---|---|
| AT2G46830 | CCA1 | | [3, 4] |
| AT2G47070 | SPL1 | Age regulated | [1] |
| AT3G04610 | FLK | Autonomous | [1] |
| AT3G05120 | GID1A | GA | [1] |
| AT3G10390 | FLD | Autonomous | [1] |
| AT3G11540 | SPY | GA | [1] |
| AT3G12810 | PIE1 | | [4, 12] |
| AT3G15270 | SPL5 | | [2] |
| AT3G15354 | SPA3 | Photoperiod | [1] |
| AT3G18990 | VRN1 | Vernalization | [1] |
| AT3G19140 | DNF | Photoperiod | [1] |
| AT3G24440 | VIL1 | Vernalization | [5] |
| AT3G33520 | ARP6 | Ambient temperature | [1] |
| AT3G44680 | HDA9 | | [13] |
| AT3G48430 | REF6 | Vernalization | [14] |
| AT3G54340 | AP3 | | [15] |
| AT3G54990 | SMZ | Photoperiod | [1] |
| AT3G63010 | GID1B | GA | [1] |
| AT4G00450 | CCT | | [16, 17] |
| AT4G00650 | FRI | Vernalization | [1] |
| AT4G02560 | LD | Autonomous | [1] |
| AT4G02780 | GA1 | GA | [1] |
| AT4G08920 | CRY2 | Photoperiod | [1] |
| AT4G11110 | SPA2 | Photoperiod | [1] |
| AT4G16280 | FCA | Autonomous | [1] |
| AT4G16845 | VRN2 | Vernalization | [1] |
| AT4G20370 | TSF | Integrator | [12] |
| AT4G22950 | AGL19 | | [5] |
| AT4G24540 | AGL24 | Integrator | [1, 2] |
| AT4G25530 | FWA | | [10] |
| AT4G26150 | GNL | GA | [1] |
| AT4G30972 | MIR156B | Age regulated | [1] |
| AT4G31877 | MIR156C | Age regulated | [1] |
| AT4G32551 | LEU | | [6] |
| AT4G34530 | CIB1 | | [2] |
| AT4G35900 | FD | Photoperiod | [1, 2, 9] |
| AT4G36920 | AP2 | | [1] |
| AT5G03790 | LMI1 | | [18] |
| AT5G03840 | TFL1 | | [6, 2] |
| AT5G04275 | MIR172B | Photoperiod | [1] |
| AT5G06850 | FTIP1 | | [19] |
| AT5G10140 | FLC | Autonomous | [1] |
| AT5G10945 | MIR156D | Age regulated | [1] |
| AT5G11977 | MIR156E | Age regulated | [1] |
| AT5G13480 | FY | Autonomous | [1] |
| AT5G15160 | FLX | | [8] |
| AT5G15840 | CO | Photoperiod | [1] |
| AT5G16320 | FRI1 | Vernalization | [15] |
| AT5G17690 | LHP1 | Vernalization | [1] |
| AT5G17690 | TFL2 | | [4] |
| AT5G26147 | MIR156F | Age regulated | [1] |

Continued

| AGI code | Gene name | Pathway | Reference |
|---|---|---|---|
| AT5G27320 | GID1C | GA | [1] |
| AT5G35840 | PHYC | Photoperiod | [4] |
| AT5G37055 | SEF | | [20] |
| AT5G48890 | LATE | | [21] |
| AT5G51230 | EMF2 | | [1] |
| AT5G56860 | GNC | GA | [1] |
| AT5G57380 | VIN3 | Vernalization | [1] |
| AT5G60120 | TOE2 | | [1, 2] |
| AT5G60910 | FUL | | [6, 2] |
| AT5G61380 | TOC1 | | [4, 3] |
| AT5G61850 | LFY | Integrator | [1, 18] |
| AT5G61920 | FLX4 | | [8] |
| AT5G62430 | CDF1 | Photoperiod | [1] |
| AT5G67180 | TOE3 | | [1] |

# References


[1] Srikanth A, Schmid M. Regulation of flowering time: all roads lead to Rome. Cellular and Molecular Life Sciences. 2011;68(12):2013–2037.

[2] Andres F, Coupland G. The genetic basis of flowering responses to seasonal cues. Nat Rev Genet. 2012;13(9):627–39.

[3] Mouradov A, Cremer F, Coupland G. Control of flowering time: Interacting pathways as a basis for diversity. Plant Cell. 2002;14:S111–S130.

[4] Putterill J, Laurie R, Macknight R. It's time to flower: the genetic control of flowering time. Bioessays. 2004;26(4):363–73.

[5] Kim DH, Doyle MR, Sung S, Amasino RM. Vernalization: Winter and the Timing of Flowering in Plants. Annual Review of Cell and Developmental Biology. 2009;25:277–299.

[6] Wellmer F, Riechmann JL. Gene networks controlling the initiation of flower development. Trends in Genetics. 2010;26(12):519–527.

[7] Magome H, Yamaguchi S, Hanada A, Kamiya Y, Oda K. The DDF1 transcriptional activator upregulates expression of a gibberellin-deactivating gene, GA2ox7, under high-salinity stress in Arabidopsis. Plant J. 2008;56(4):613–26.

[8] Ding L, Kim SY, Michaels SD. FLOWERING LOCUS C EXPRESSOR family proteins regulate FLOWERING LOCUS C expression in both winter-annual and rapid-cycling Arabidopsis. Plant Physiol. 2013;163(1):243–52.

[9] Jaeger KE, Pullen N, Lamzin S, Morris RJ, Wigge PA. Interlocking feedback loops govern the dynamic behavior of the floral transition in Arabidopsis. Plant Cell. 2013;25(3):820–33.

[10] Komeda Y. Genetic regulation of time to flower in Arabidopsis thaliana. Annual Review of Plant Biology. 2004;55:521–535.

[11] Schmitz RJ, Hong L, Michaels S, Amasino RM. FRIGIDA-ESSENTIAL 1 interacts genetically with FRIGIDA and FRIGIDA-LIKE 1 to promote the winter-annual habit of Arabidopsis thaliana. Development. 2005;132(24):5471–5478.

[12] Flowers JM, Hanzawa Y, Hall MC, Moore RC, Purugganan MD. Population Genomics of the Arabidopsis thaliana Flowering Time Gene Network. Molecular Biology and Evolution. 2009;26(11):2475–2486.

[13] Zografos BR, Sung SB. Vernalization-mediated chromatin changes. Journal of Experimental Botany. 2012;63(12):4343–4348.

[14] Hou X, Zhou J, Liu C, Liu L, Shen L, Yu H. Nuclear factor Y-mediated H3K27me3 demethylation of the SOC1 locus orchestrates flowering responses of Arabidopsis. Nat Commun. 2014;5:4601.

[15] Alvarez-Buylla ER, Benitez M, Corvera-Poire A, Chaos Cador A, de Folter S, Gamboa de Buen A, et al. Flower development. Arabidopsis Book. 2010;8:e0127.

[16] Imura Y, Kobayashi Y, Yamamoto S, Furutani M, Tasaka M, Abe M, et al. Cryptic Precocious/Med12 Is a Novel Flowering Regulator with Multiple Target Steps in Arabidopsis. Plant and Cell Physiology. 2012;53(2):287–303.

[17] Gillmor CS, Silva-Ortega CO, Willmann MR, Buendia-Monreal M, Poethig RS. The Arabidopsis Mediator CDK8 module genes CCT (MED12) and GCT (MED13) are global regulators of developmental phase transitions. Development. 2014;141(23):4580–4589.

[18] Saddic LA, Huvermann BR, Bezhani S, Su YH, Winter CM, Kwon CS, et al. The LEAFY target LMI1 is a meristem identity regulator and acts together with LEAFY to regulate expression of CAULIFLOWER. Development. 2006;133(9):1673–1682.

[19] Liu L, Liu C, Hou XL, Xi WY, Shen LS, Tao Z, et al. FTIP1 Is an Essential Regulator Required for Florigen Transport. Plos Biology. 2012;10(4).

[20] March-Diaz R, Garcia-Dominguez M, Florencio FJ, Reyes JC. SEF, a new protein required for flowering repression in Arabidopsis, interacts with PIE1 and ARP6. Plant Physiology. 2007;143(2):893–901.

[21] Weingartner M, Subert C, Sauer N. LATE, a C2H2 zinc-finger protein that acts as floral repressor. Plant Journal. 2011;68(4):681–692.
