# Supplementary material for: "Missing" G x E Variation Controls Flowering Time in Arabidopsis thaliana
Source: PLoS Genet. 2015 Oct 16;11(10):e1005597. doi: 10.1371/journal.pgen.1005597 (PMC4608753; doi:10.1371/journal.pgen.1005597)
Supplement: S4 Table — (PDF) [file pgen.1005597.s011.pdf]

Table S4. All associations significant at the 10^{-5} level.

Chr	Position	MAF	*p*-value	Gene ID	Distaince (bp)	Description
Full SNP test						
5	3180721	39.1	2.72E-09	AT5G10140	1273	FLC;MADS-box transcription factor family protein
				AT5G10150	1262	Domain of unknown function (DUF966)
2	9005735	39.7	2.33E-08	AT2G20950	0	Arabidopsis phospholipase-like protein (PEARLI 4) family
2	9038659	36.8	5.83E-08	AT2G21060	1009	ATCSP4;glycine-rich protein 2B
				AT2G21070	2193	FIO1;methyltransferases
2	9002939	39.7	8.18E-08	AT2G20940	238	Protein of unknown function (DUF1279)
				AT2G20950	196	Arabidopsis phospholipase-like protein (PEARLI 4) family
2	9034914	25.9	1.06E-07	AT2G21050	0	LAX2;like AUXIN RESISTANT 2
2	9041551	39.1	1.96E-07	AT2G21070	0	FIO1;methyltransferases
2	9046018	39.1	1.96E-07	AT2G21090	0	Pentatricopeptide repeat (PPR-like) superfamily protein
2	9049645	39.1	1.96E-07	AT2G21100	241	Disease resistance-responsive family protein
				AT2G21110	645	Disease resistance-responsive family protein
2	9052359	39.1	1.96E-07	AT2G21120	0	Protein of unknown function (DUF803)
2	9044852	38.5	2.59E-07	AT2G21080	0	protein coding
2	9047636	38.5	2.59E-07	AT2G21090	57	Pentatricopeptide repeat (PPR-like) superfamily protein
				AT2G21100	495	Disease resistance-responsive family protein
2	9049040	38.5	2.59E-07	AT2G21100	0	Disease resistance-responsive family protein
2	9050568	38.5	2.59E-07	AT2G21110	0	Disease resistance-responsive family protein
2	9050927	38.5	2.59E-07	AT2G21110	77	Disease resistance-responsive family protein
				AT2G21120	821	Protein of unknown function (DUF803)
2	9001385	33.3	3.82E-07	AT2G20930	0	SNARE-like superfamily protein
2	9001740	33.3	3.82E-07	AT2G20930	3	SNARE-like superfamily protein
				AT2G20940	292	Protein of unknown function (DUF1279)
5	3184162	29.3	5.54E-07	AT5G10150	297	Domain of unknown function (DUF966)
				AT5G10160	1584	Thioesterase superfamily protein
5	17742294	21.8	9.13E-07	AT5G44080	2421	bZIP transcription factor family protein
				AT5G44090	472	Calcium-binding EF-hand family protein
2	8989557	32.8	9.81E-07	AT2G20900	0	ATDGK5,DGK5;diacylglycerol kinase 5
5	3358289	32.8	1.10E-06	AT5G10620	1674	methyltransferases
				AT5G10625	498	protein coding
2	9070665	36.2	2.68E-06	AT2G21160	234	Translocon-associated protein, alpha subunit
				AT2G21170	89	PDTPI;triosephosphate isomerase
2	9593397	25.9	2.96E-06	AT2G22590	0	protein coding
2	9596991	25.9	2.96E-06	AT2G22600	0	RNA-binding KH domain-containing protein
2	8778734	35.1	4.66E-06	AT2G20330	3120	Transducin/WD40 repeat-like superfamily protein
				AT2G20340	1001	PLP-dependent transferases superfamily protein
2	9151447	33.9	5.28E-06	AT2G21385	0	protein coding
2	9037421	24.7	5.30E-06	AT2G21060	0	ATCSP4;glycine-rich protein 2B
5	3748839	42.5	5.40E-06	AT5G11650	1714	alpha/beta-Hydrolases superfamily protein
				AT5G11660	41	Protein of Unknown Function (DUF239)
2	9091028	38.5	5.61E-06	AT2G21220	1215	SAUR-like auxin-responsive protein family
				AT2G21230	2596	bZIP transcription factor family protein
5	3359368	35.1	6.62E-06	AT5G10625	0	protein coding
2	9036673	23.6	7.50E-06	AT2G21050	37	LAX2;like AUXIN RESISTANT 2
				AT2G21060	120	ATCSP4;glycine-rich protein 2B
2	8640484	28.2	7.79E-06	AT2G20010	0	Protein of unknown function (DUF810)
5	19897840	32.2	8.06E-06	AT5G49100	0	protein coding
2	9589040	24.7	8.11E-06	AT2G22560	202	Kinase interacting (KIP1like) family protein

Continued

Chr	Position	MAF	p-value	Gene ID	Distaince (bp)	Description
				AT2G22570	395	ATNIC1;nicotinamidase 1
2	9590291	24.7	8.11E-06	AT2G22570	0	ATNIC1;nicotinamidase 1
5	23249568	10.9	8.48E-06	AT5G57380	64	VIN3;Fibronectin type III domain-containing protein
				AT5G57390	3891	AIL5;AINTEGUMENTA-like 5
2	9145587	44.8	8.81E-06	AT2G21380	0	Kinesin motor family protein
Common SNP effects						
2	9005735	39.7	3.68E-09	AT2G20950	0	Arabidopsis phospholipase-like protein (PEARLI 4) family
2	9038659	36.8	1.01E-08	AT2G21060	1009	ATCSP4,ATGRP2B,GRP2B;glycine-rich protein 2B
				AT2G21070	2193	FIO1;methyltransferases
2	9002939	39.7	1.23E-08	AT2G20940	238	Protein of unknown function (DUF1279)
				AT2G20950	196	Arabidopsis phospholipase-like protein (PEARLI 4) family
2	9034914	25.9	2.84E-08	AT2G21050	0	LAX2;like AUXIN RESISTANT 2
2	9041551	39.1	3.47E-08	AT2G21070	0	FIO1;methyltransferases
2	9046018	39.1	3.47E-08	AT2G21090	0	Pentatricopeptide repeat (PPR-like) superfamily protein
2	9049645	39.1	3.47E-08	AT2G21100	241	Disease resistance-responsive family protein
				AT2G21110	645	Disease resistance-responsive family protein
2	9052359	39.1	3.47E-08	AT2G21120	0	Protein of unknown function (DUF803)
2	9044852	38.5	3.97E-08	AT2G21080	0	protein coding
2	9047636	38.5	3.97E-08	AT2G21090	57	Pentatricopeptide repeat (PPR-like) superfamily
				AT2G21100	495	Disease resistance-responsive family protein
2	9049040	38.5	3.97E-08	AT2G21100	0	Disease resistance-responsive family protein
2	9050568	38.5	3.97E-08	AT2G21110	0	Disease resistance-responsive family protein
2	9050927	38.5	3.97E-08	AT2G21110	77	Disease resistance-responsive family protein
				AT2G21120	821	Protein of unknown function (DUF803)
2	9001385	33.3	6.13E-08	AT2G20930	0	SNARE-like superfamily protein
2	9001740	33.3	6.13E-08	AT2G20930	3	SNARE-like superfamily protein
				AT2G20940	292	Protein of unknown function (DUF1279)
5	17742294	21.8	1.38E-07	AT5G44080	2421	bZIP transcription factor family protein
				AT5G44090	472	Calcium-binding EF-hand family protein
2	8989557	32.8	1.67E-07	AT2G20900	0	ATDGK5;diacylglycerol kinase 5
5	3180721	39.1	3.58E-07	AT5G10140	1273	FLC; MADS-box transcription factor family protein
				AT5G10150	1262	Domain of unknown function (DUF966)
2	9070665	36.2	4.12E-07	AT2G21160	234	Translocon-associated protein (TRAP), alpha subunit
				AT2G21170	89	PDTPI;triosephosphate isomerase
2	8778734	35.1	7.47E-07	AT2G20330	3120	Transducin/WD40 repeat-like superfamily protein
				AT2G20340	1001	PLP-dependent transferases superfamily protein
2	9091028	38.5	9.27E-07	AT2G21220	1215	SAUR-like auxin-responsive protein family
				AT2G21230	2596	bZIP transcription factor family protein
5	23249568	10.9	1.47E-06	AT5G57380	64	VIN3;Fibronectin type III domain-containing protein
				AT5G57390	3891	AIL5;AINTEGUMENTA-like 5
2	9149839	45.4	1.84E-06	AT2G21385	0	protein coding
2	9145587	44.8	2.08E-06	AT2G21380	0	Kinesin motor family protein
2	9014510	50	2.18E-06	AT2G20980	579	MCM10;minichromosome maintenance 10
				AT2G20990	42	ATSYTA;synaptotagmin A
2	9002083	39.1	2.19E-06	AT2G20940	0	Protein of unknown function (DUF1279)
5	23543157	12.1	2.26E-06	AT5G58170	0	GDPDL7;SHV3-like 5
2	9154645	73.6	2.28E-06	AT2G21390	0	Coatomer, alpha subunit
2	9156966	73.6	2.28E-06	AT2G21390	88	Coatomer, alpha subunit
				AT2G21400	1424	SRS3;SHI-related sequence3

Continued

Chr	Position	MAF	*p*-value	Gene ID	Distaince (bp)	Description
2	9017941	28.7	2.38E-06	AT2G20990	0	ATSYTA;synaptotagmin A
2	9027894	28.7	2.38E-06	AT2G21045	0	Rhodanese/Cell cycle control phosphatase superfamily protein
2	9037421	75.3	2.40E-06	AT2G21060	0	ATCSP4;glycine-rich protein 2B
5	23534278	10.3	2.56E-06	AT5G58160	0	actin binding
2	9029072	33.9	2.56E-06	AT2G21045	52	Rhodanese/Cell cycle control phosphatase superfamily protein
				AT2G21050	5018	LAX2;like AUXIN RESISTANT 2
2	9036673	23.6	2.63E-06	AT2G21050	37	LAX2;like AUXIN RESISTANT 2
				AT2G21060	120	ATCSP4,ATGRP2B,GRP2B;glycine-rich protein 2B
5	23544472	11.5	2.84E-06	AT5G58180	0	ATYKT62;Synaptobrevin family protein
5	23545876	11.5	2.84E-06	AT5G58180	116	ATYKT62;Synaptobrevin family protein
				AT5G58190	453	ECT10;evolutionarily conserved C-terminal region 10
5	23547556	11.5	2.84E-06	AT5G58190	0	ECT10;evolutionarily conserved C-terminal region 10
5	23550180	11.5	2.84E-06	AT5G58200	0	Calcineurin-like metallo-phosphoesterase superfamily protein
5	23560954	11.5	2.84E-06	AT5G58260	0	NdhN;oxidoreductases
5	23530884	9.8	3.05E-06	AT5G58140	0	NPL1;phototropin 2
2	9060886	35.6	3.81E-06	AT2G21140	0	ATPRP2;proline-rich protein 2
4	7065838	90.8	4.47E-06	AT4G11720	0	GCS1;hapless 2
4	17599693	28.7	4.60E-06	AT4G37432	1003	other RNA
				AT4G37440	1529	protein coding
2	8795687	21.3	4.65E-06	AT2G20380	0	Galactose oxidase/kelch repeat superfamily protein
2	8796100	21.3	4.65E-06	AT2G20380	120	Galactose oxidase/kelch repeat superfamily protein
				AT2G20390	183	protein coding
2	9088650	22.4	5.16E-06	AT2G21210	2741	SAUR-like auxin-responsive protein family
				AT2G21220	676	SAUR-like auxin-responsive protein family
2	9149629	48.3	5.85E-06	AT2G21380	320	Kinesin motor family protein
				AT2G21385	10	protein coding
5	23177309	20.1	6.34E-06	AT5G57190	1945	PSD2;phosphatidylserine decarboxylase 2
				AT5G57200	387	ENTH/ANTH/VHS superfamily protein
5	23140522	12.6	6.39E-06	AT5G57126	1116	transposable element gene
				AT5G57130	4458	Clp amino terminal domain-containing protein
3	5255916	73.6	6.48E-06	AT3G15530	1354	SAM MTases superfamily protein
				AT3G15534	2960	protein coding
2	8892926	58	6.96E-06	AT2G20620	318	Protein of unknown function (DUF626)
2	9596991	74.1	7.36E-06	AT2G22600	374	RNA-binding KH domain-containing protein
5	23587485	9.8	7.63E-06	AT5G58350	0	WNK4;with no lysine (K) kinase 4
5	23592035	9.8	7.63E-06	AT5G58360	1066	ATOFP3;ovate family protein 3
				AT5G58370	1082	Ploop_NTPase
2	8785331	21.8	8.28E-06	AT2G20350	0	Integrase-type DNA-binding superfamily protein
3	19403042	24.7	8.42E-06	AT3G52320	0	F-box and associated interaction domains-containing protein
5	23111982	24.7	8.56E-06	AT5G57110	0	ACA8;autoinhibited Ca2+ -ATPase, isoform 8
2	11276755	24.7	8.89E-06	AT2G26510	0	PDE135;Xanthine/uracil permease family protein
2	9589040	24.7	8.92E-06	AT2G22560	202	Kinase interacting (KIP1-like) family protein
				AT2G22570	395	ATNIC1;nicotinamidase 1
2	9590291	24.7	8.92E-06	AT2G22570	0	ATNIC1;nicotinamidase 1

G_{SNP} x E effects

NA
