# Supplementary material for: "Missing" G x E Variation Controls Flowering Time in Arabidopsis thaliana
Source: PLoS Genet. 2015 Oct 16;11(10):e1005597. doi: 10.1371/journal.pgen.1005597 (PMC4608753; doi:10.1371/journal.pgen.1005597)
Supplement: S5 Table — (PDF) [file pgen.1005597.s012.pdf]

Table S5. Summary of associations in MTMM and VCA.

Cluster	Cluster name	Start	End	VCA (LR)			GWAS (*p*-value)			Candidate genes	
				Full local	G_{local}	G_{local} x E	Full SNP	Common	G_{SNP} x E	Genes in *a priori* list	Genes in GO annotationt
1	Chr1_596741_663259	AT1G02840	AT1G02850	**5.47**	-0.01	**5.48**	1.23E-04	1.90E-01	2.73E-05		
2	Chr1_963400_1053719	AT1G03905	AT1G03982	**7.36**	0.03	**7.33**	3.27E-03	6.68E-02	1.17E-03		
3	Chr1_29729157_29812622	AT1G79110	AT1G79170	**5.71**	**5.25**	1.1	8.99E-04	6.91E-04	1.26E-02		
4	Chr2_8597699_8684450	AT2G19980	AT2G20050	3.21	3.17	0.51	**7.79E-06**	4.12E-04	5.08E-04		
5	Chr2_8736739_8843644	AT2G20320	AT2G20440	3.97	3.93	0.13	**4.66E-06**	**7.47E-07**	1.20E-02		AT2G20330
6	Chr2_8847960_8937339	AT2G20610	AT2G20650	2.56	2.56	0.12	4.10E-05	**6.96E-06**	4.11E-03		
7	Chr2_8941712_9203339	AT2G20840	AT2G21430	**13.49**	**13.49**	2.97	**2.33E-08**	**3.68E-09**	2.06E-03		*FIO1*, AT2G21150
8	Chr2_9288258_9350002	AT2G21860	AT2G21860	**5.15**	**5.14**	0	7.18E-05	5.62E-05	1.21E-02		
9	Chr2_9542796_9640595	AT2G22530	AT2G22620	3.91	2.48	1.51	**2.96E-06**	**7.36E-06**	8.99E-05	*SVP*	AT2G22610
10	Chr2_9627344_9703928	AT2G22720	AT2G22750	**5.89**	2.21	4.01	4.07E-05	3.40E-04	7.96E-04		
11	Chr2_9814299_9896562	AT2G23130	AT2G23170	**6.05**	0.91	**5.75**	2.48E-03	4.03E-03	4.88E-03		
12	Chr2_10741694_10809226	AT2G25300	AT2G25310	**5.41**	4.18	1.37	4.12E-05	1.26E-05	4.89E-03		
13	Chr2_11229153_11323445	AT2G26470	AT2G26550	2.47	1.98	0.79	5.05E-05	**8.89E-06**	1.36E-02		
14	Chr2_17978482_18061243	AT2G43350	AT2G43410	**5.45**	0.04	**5.4**	5.67E-03	1.01E-02	1.93E-03	*FPA*	AT2G43370
15	Chr3_1709981_1773163	AT3G05840	AT3G05840	**5.08**	0.01	**5.07**	1.56E-02	2.17E-02	5.25E-03		
16	Chr3_5213510_5301922	AT3G15510	AT3G15570	3.36	3.36	0	1.81E-05	**6.48E-06**	3.13E-03		
17	Chr3_5509788_5588589	AT3G16340	AT3G16370	**5.56**	**5.54**	0.17	1.13E-03	2.55E-04	1.99E-03		
18	Chr3_5569778_5735313	AT3G16480	AT3G16760	**6.66**	4.95	3.17	4.41E-05	4.17E-05	1.13E-04		
19	Chr3_5729375_5807889	AT3G16860	AT3G16920	**6.46**	1.36	5.1	1.44E-03	8.34E-04	8.82E-03		
20	Chr3_5795289_5855867	AT3G17080	AT3G17080	**5.45**	2.63	2.83	7.76E-04	1.61E-03	2.80E-03		
21	Chr3_5890399_5976466	AT3G17340	AT3G17360	**6.17**	3.71	2.79	1.84E-05	8.39E-05	2.13E-03		
22	Chr3_5949834_6015993	AT3G17470	AT3G17490	**5.3**	**5.3**	0	5.75E-05	3.06E-04	6.71E-03		
23	Chr3_17073173_17167874	AT3G46480	AT3G46550	**6.96**	0	**6.96**	1.32E-04	1.04E-02	3.39E-05		
24	Chr3_19357180_19449782	AT3G52270	AT3G52370	0	-0.01	0.02	2.16E-05	**8.42E-06**	1.15E-02		
25	Chr4_172416_285054	AT4G00450	AT4G00590	**6.37**	4.57	3.31	2.85E-04	5.12E-04	4.32E-04	*CCT*	
26	Chr4_6066725_6136607	AT4G09647	AT4G09660	**6**	0	**6**	1.98E-03	6.80E-03	1.08E-03		
27	Chr4_10233969_10295439	AT4G18650	AT4G18650	**5.12**	0.12	**5.0**	1.57E-02	3.89E-02	1.28E-02		
28	Chr4_11410936_11471734	AT4G21500	AT4G21500	**5**	0.2	4.81	6.08E-03	8.17E-02	7.23E-03		
29	Chr4_17554051_17645776	AT4G37400	AT4G37460	2.83	2.83	0	2.29E-05	**4.60E-06**	7.54E-04		
30	Chr5_3123722_3251515	AT5G10090	AT5G10260	**11.69**	**9.65**	4.1	**2.72E-09**	**3.58E-07**	9.85E-05	*FLC*	
31	Chr5_3313593_3407451	AT5G10572	AT5G10690	4.67	2.25	2.59	1.10E-06	6.16E-04	2.64E-06		
32	Chr5_3688810_3801416	AT5G11580	AT5G11700	**6.45**	4.66	2.16	5.40E-06	3.44E-04	7.17E-04		
33	Chr5_17400539_17463009	AT5G43403	AT5G43405	**5.34**	**5.3**	0.04	4.34E-05	9.22E-05	1.01E-02		
34	Chr5_17701005_17788288	AT5G44060	AT5G44120	2.28	1.83	0.6	**9.13E-07**	**1.38E-07**	1.97E-03		
35	Chr5_19844944_19939399	AT5G49030	AT5G49120	1.35	-0.01	1.37	8.06E-06	6.83E-03	5.88E-05		
36	Chr5_22308672_22440944	AT5G55045	AT5G55250	**6.95**	2.37	4.89	1.25E-05	4.73E-03	2.24E-06		
37	Chr5_23066174_23221617	AT5G57070	AT5G57230	**6.03**	**6.03**	0.06	3.25E-05	**6.34E-06**	9.86E-03		AT5G57130
38	Chr5_23199407_23297883	AT5G57345	AT5G57410	**6.31**	**6.31**	0.1	**8.48E-06**	**1.47E-06**	6.76E-03	*VIN3*	
39	Chr5_23452187_23637028	AT5G58005	AT5G58400	**8.51**	**8.51**	0.01	1.37E-05	**2.26E-06**	8.83E-03		
	Number of regions			28	10	8	7	13	0		
	Number of SNPs			43554	16063	11125	144	238	0		
	Total regions (%)			2.00	0.88	0.47	NA	NA	NA		
